# Acetate sensing by GPR43 alarms neutrophils and protects from severe sepsis

**DOI:** 10.1038/s42003-021-02427-0

**Published:** 2021-07-30

**Authors:** Katja Schlatterer, Christian Beck, Ulrich Schoppmeier, Andreas Peschel, Dorothee Kretschmer

**Affiliations:** 1 Interfaculty Institute for Microbiology and Infection Medicine Tübingen (IMIT), Infection Biology, University of Tübingen, Tübingen, Germany; 2 German Center for Infection Research, partner site Tübingen, Tübingen, Germany; 3 Cluster of Excellence EXC 2124 Controlling Microbes to Fight Infections, Tübingen, Germany; 4 Interfaculty Institute for Microbiology and Infection Medicine Tübingen (IMIT), Medical Microbiology and Hygiene, University of Tübingen, Tübingen, Germany

**Keywords:** Bacterial infection, Bacterial infection

## Abstract

Bacterial sepsis is a major cause of mortality resulting from inadequate immune responses to systemic infection. Effective immunomodulatory approaches are urgently needed but it has remained elusive, which targets might be suitable for intervention. Increased expression of the G-protein-coupled receptor GPR43, which is known to govern intestinal responses to acetate, has been associated with sepsis patient survival but the mechanisms behind this observation have remained unclear. We show that elevated serum acetate concentrations prime neutrophils in a GPR43-dependent fashion, leading to enhanced neutrophil chemotaxis, oxidative burst, cytokine release and upregulation of phagocytic receptors. Consequently, acetate priming improved the capacity of human neutrophils to eliminate methicillin-resistant *Staphylococcus aureus*. Acetate administration increased mouse serum acetate concentrations and primed neutrophils. Notably, it rescued wild-type mice from severe *S. aureus* sepsis and reduced bacterial numbers in peripheral organs by several magnitudes. Acetate treatment improved the sepsis course even when applied several hours after onset of the infection, which recommends GPR43 as a potential target for sepsis therapy. Our study indicates that the severity of sepsis depends on transient neutrophil priming by appropriate blood acetate concentrations. Therapeutic interventions based on GPR43 stimulation could become valuable strategies for reducing sepsis-associated morbidity and mortality.

## Abstract (web summary)

Katja Schlatterer et al. use mouse models to show that elevated serum acetate concentrations prime human neutrophils in a GPR43-dependent fashion, and rescue mice from severe sepsis. These results suggest microbiome-, diet-, or pathogen-derived short-chain fatty acids govern the defense capacities of immune cells, potentially hinting at the therapeutic potential of GPR43 in treating sepsis.

## Introduction

Bacterial infections represent a major cause for severe diseases whose therapy is complicated by worldwide increasing rates of antibiotic resistance^1^. Disseminated bacterial bloodstream infections represent a frequent complication, leading to life-threatening sepsis and septic shock with multi-organ failure^2^. Sepsis is a common reason for intensive-care unit admission also in high-income countries, causing for instance 750,000 cases per year with an estimated death rate of about 30% in the United States^2,3^. The bacterial pathogen *Staphylococcus aureus* is one of the most frequent causative agents of sepsis^4,5^. Many of these infections are caused by methicillin-resistant *S. aureus* (MRSA) strains, which can be treated only with limited efficacy by some last-resort antibiotics^6^. Sepsis-related pathology results from insufficient or dysregulated immune responses involving multiple immune cells and signaling pathways, the complex interplay of which limits our understanding and the development of effective preventive or therapeutic interventions^7^. Accordingly, the therapy of sepsis has not made major progress in the last decades and new approaches that modulate systemic immune responses in suitable ways are urgently needed^8^.

Bacterial infections are primarily contained by neutrophil granulocytes, potent phagocytic cells, and the most abundant leukocytes in the bloodstream^9,10^. Neutrophils express various well-studied pattern recognition receptors including Toll-like receptors (TLRs)^11^ and formyl-peptide receptors (FPRs)^12^ on their surface, in order to detect ‘microbe-associated molecular pattern’ (MAMP) molecules, hallmark signals for invasive infections^13^. FPRs belong to the family of G-protein coupled receptors, members of which sense for instance chemokines or bacterial products such as formylated peptides^12^ and phenol-soluble modulin peptides^14–16^. Stimulation of various GPCR can establish a ‘primed’ state, which allows neutrophils to trigger a maximal antimicrobial response upon further pro-inflammatory stimulation^17^. The role of neutrophil priming and activation in sepsis remains elusive—activated neutrophils are essential for pathogen elimination, in particular by the release of reactive oxygen species (ROS), but the exuberant and prolonged release of ROS can contribute to multi-organ failure^18,19^.

Neutrophils also express GPR43, a GPCR that recognizes the short-chain fatty acids (SCFA) acetate, propionate, and butyrate^20^. GPR43 is expressed for instance on enterocytes^21^ and is known to have a critical role in monitoring intestinal SCFA levels with critical consequences for chronic metabolic and inflammatory disorders such as obesity, gout, or colitis^22^. In contrast, far less is known about the consequences of GPR43 activation in neutrophils. Increased expression of GPR43 on blood cells is linked to enhanced survival of septic patients^23^, strongly suggesting that this receptor plays a critical role in the host defense against systemic infections. However, if and how GPR43 activation of neutrophils may influence the outcome of severe sepsis has remained unclear. Among the potential GPR43 agonists, only acetate can reach concentrations in human serum that would be sufficient to activate GPR43^24,25^. However, serum acetate levels vary strongly according to individual nutritional and metabolic properties (0.02–2 mM)^24,26^ with an average concentration of ca. 0.050^27^ and it remains elusive under which conditions GPR43 may prime or activate neutrophils or not. Moreover, the acetate concentrations required for activation of GPR43 are high enough to affect the pH of culture media and the functions of human cells, which may have contributed to inconsistent reports about either pro- or anti-inflammatory roles of GPR43 in neutrophils^28,29^.

We demonstrate that GPR43 can prime neutrophils to enhance their capacity to eliminate bacterial pathogens. Average serum acetate concentrations were not sufficient to achieve full priming, but interventional acetate injection led to strongly improved capacities of mice to cure *S. aureus* infections in a GPR43-dependent fashion, even when applied several hours after onset of the infection.

## Results

### GPR43 activation by acetate primes neutrophils

**Fig. 1:**
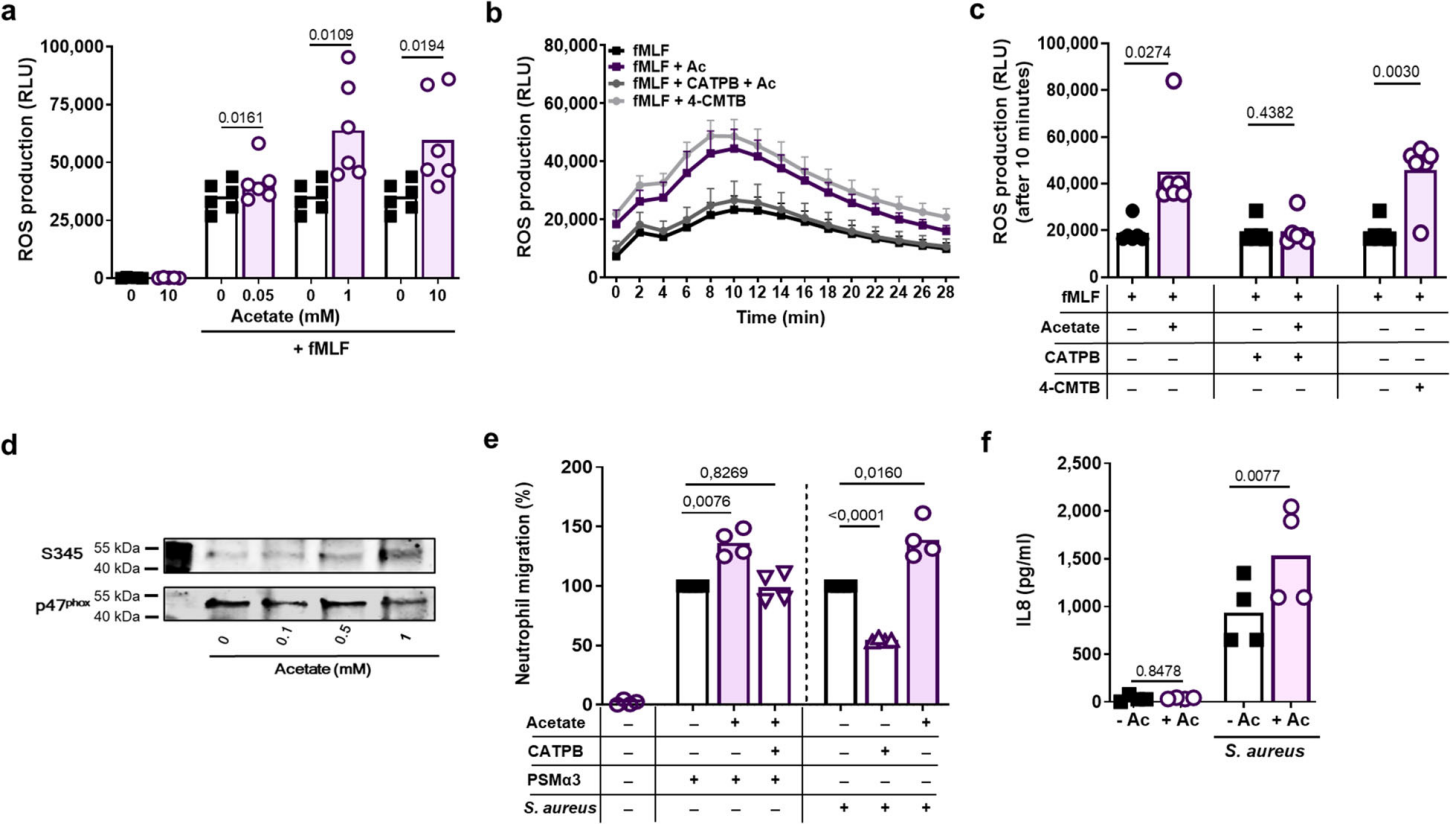
Acetate primes neutrophils in a GPR43-dependent manner. Acetate enhanced the oxidative burst induced by (**a**) the FPR1 ligand fMLF (500 nM), which could be inhibited by the GPR43 antagonist CATPB (**b**). The same effect was caused by the synthetic GPR43 ligand 4-CMTB. ROS production was monitored by measuring relative luminescence units (RLU) emitted by ROS-responsive luminol (**c**). Incubation of neutrophils with acetate induced the priming-associated phosphorylation of the NADPH oxidase subunit p47^phox^ at serine position 345 (S345) (**d**). Migration of neutrophils towards the bacteria-derived chemoattractant FPR2 ligand PSMα3 (500 nM) or towards live *S. aureus* cells (**e**), as well as release of the chemokine IL-8 in response to *S. aureus* challenge (both MOI of 1) were enhanced upon neutrophil priming with 1 mM acetate (Ac) (**f**). Data in panels **a**, **c**, **e** and **f** represent means of *n* = 6 (**a**, **c**) and *n* = 4 (**e**, **f**) independent experiments. Data in panel **b** represent mean ± SEM of *n* = 7 independent experiments. *P < 0.05; **P < 0.01 ***P < 0.001, significant difference versus the indicated or non-acetate treated control (0 or - Ac) as calculated by paired two-tailed Student’s *t* test (**a**, **c**, **f**) or one-way ANOVA with Dunnett’s multiple comparisons test (**e**). **d** Shows one representative experiment out of three.

GPR43 is highly expressed on the surface of neutrophils^20^, suggesting a role in infection control. An essential feature of the host defence of neutrophils is the generation of ROS, which are required for bacterial killing. To analyze if GPR43 can shape neutrophil ROS production, we monitored oxidative burst upon GPR43 activation by the natural ligand acetate or the synthetic specific agonist 4-chloro-α-(1-methylethyl)-N-2-thiazolylbenzeneacetamide) (4-CMTB)^30^. Since acetate has the capacity to alter the medium pH and thereby cause unintended activation of other receptors, we used pH-neutralized, buffered sodium acetate solutions and confirmed that the medium pH did not change. While GPR43 activation failed to induce ROS generation in the absence of other stimuli, it enhanced the oxidative burst induced by bacterial ligands of FPR1 (Fig. 1a–c and Supplementary Fig. 1a) or FPR2 (Supplementary Fig. 1a), by endogenous ligands for platelet-activating factor or C5a receptors (PAFR or C5aR, respectively) (Supplementary Fig. 1a), or by live, serum-opsonized *S. aureus* cells (Supplementary Fig. 1b, c). Acetate enhanced ROS generation at concentrations above 0.5 mM (Fig. 1a), which is far above the average but within the wide range of the reported variable human serum concentration. Serum acetate concentrations can reach up to 2 mM depending on the nutritional and metabolic status or, for instance, in the portal venous system^24,31^. The acetate-mediated effect was dependent on GPR43 as it could be completely blocked by the GPR43-specific antagonist (*S*)-3-(2-(3-chlorophenyl) acetamido)-4-(4-(trifluoromethyl)phenyl) butanoic acid (CATPB)^32^ (Fig. 1b, c, Supplementary Fig. 1a, c). Thus, GPR43 activation strongly increases the oxidative burst in neutrophils in combination with other pro-inflammatory GPCR agonists. This behavior is a hallmark of agents that can prime neutrophils and it is known to be associated with phosphorylation of the NADPH oxidase subunit p47^phox^ at serine position 345^33^. Indeed, GPR43 activation by acetate triggered p47^phox^ S345 phosphorylation (Fig. 1d, Supplementary Fig. 1d), thereby confirming that GPR43 activation primes neutrophils.

Next, we explored whether acetate sensing by GPR43 influences neutrophil migration. As for the oxidative burst, acetate alone did not induce migration of neutrophils but enhanced FPR2-dependent migration. Furthermore, neutrophil chemotaxis elicited by *S. aureus* USA200^34^ cells or by USA200 culture filtrates could be partially inhibited by CATPB, which is in agreement with the documented secretion of substantial acetate amounts by *S. aureus* (Fig. 1e, Supplementary Fig. 2a, b)^35^. Similar findings were obtained when analyzing the IL-8 cytokine secretion by neutrophils after stimulation with acetate in combination with various other GPCR or with toll-like receptor (TLR) ligands. Acetate alone failed to induce IL-8 release and did not influence IL-8RA/IL-8RB expression (Supplementary Fig. 2c) but enhanced the IL-8 secretion capacities of live serum-opsonized *S. aureus* cells or of FPR2, FPR1, TLR2, or TLR4 ligands (Fig. 1f, Supplementary Fig. 2d, e). This enhanced autocrine IL-8 release could be due to the neutrophil activation status leading to increased intracellular Ca^2+^ levels, or enhanced phagocytosis of *S. aureus*, two processes, which are known to be associated with increased IL-8 release^36,37^.

### Acetate priming augments *S. aureus* phagocytosis and killing by human neutrophils

**Fig. 2:**
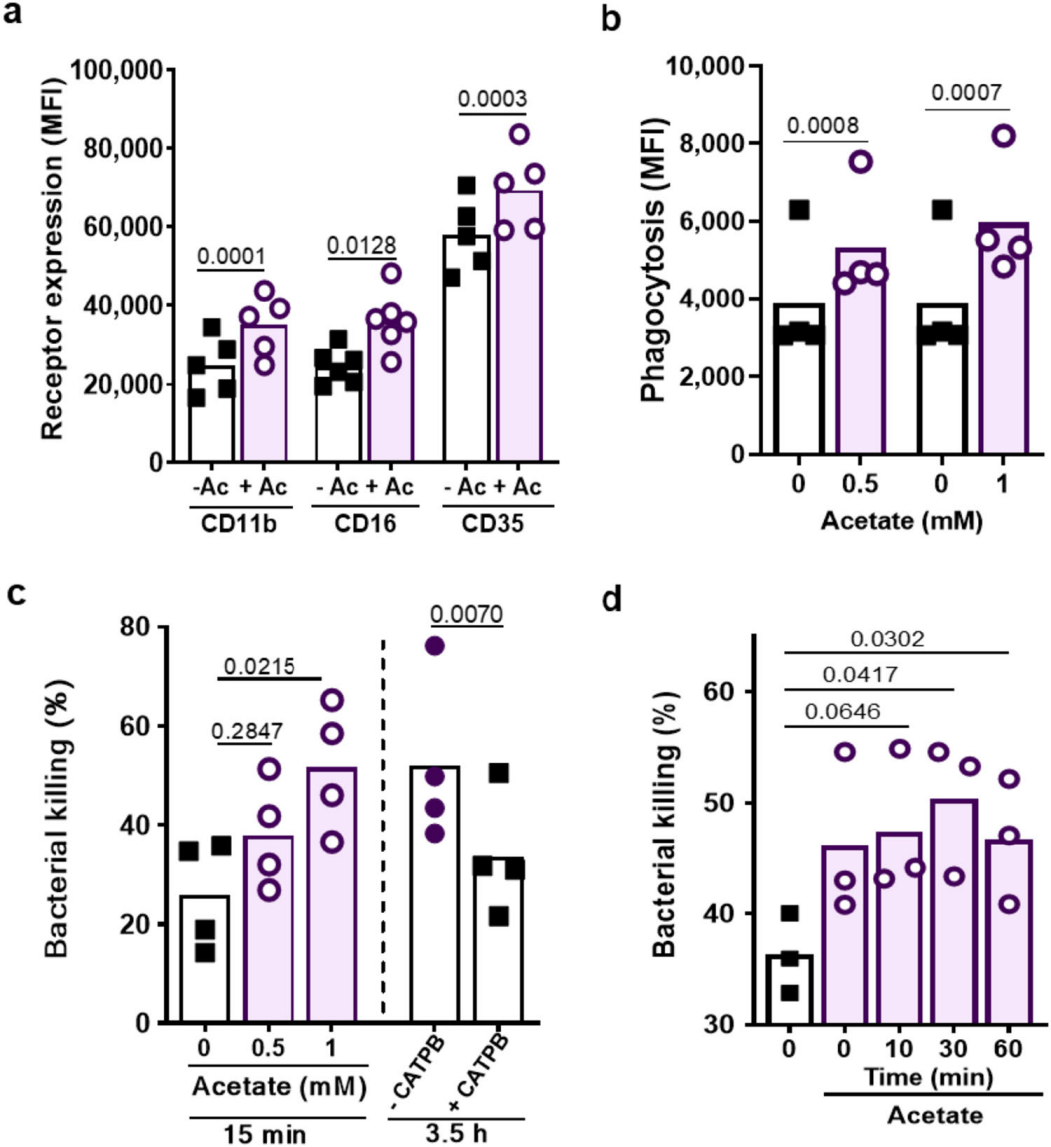
Acetate priming enhances phagocytosis and killing of *S. aureus* by human neutrophils. **a** 1 mM acetate (Ac) increased the neutrophil expression of complement (CD11b and CD35) and Fcγ (CD16) receptors given as mean fluorescence intensity (MFI) measured by flow cytometry. **b** Incubation of neutrophils for 15 min with the indicated acetate concentrations increased the phagocytic capacity as well as (**c**) the ability to kill *S. aureus* (MOI of 1) during a 15-min incubation period. Bacterial killing is expressed as dead vs. input bacterial counts (%). **c** CATPB-mediated GPR43 inhibition decreased the neutrophil capacity to kill *S. aureus* after 3.5 h co-incubation (MOI 1). **d** Addition of 1 mM acetate (Ac) at the indicated time points after the start of the *S. aureus* killing assay still improved the ability of neutrophils to kill bacteria. Data in panels **b** and **c** represent means from *n* = 4, in panel a means from *n* = 5 and in panel d means from *n* = 3 independent experiments. *P < 0.05; **P < 0.01 ***P < 0.001, significant difference versus the indicated condition as calculated by the paired two-tailed Student’s *t* test (**a**, **b**), or one way ANOVA with Dunnett’s multiple comparisons test (**c** and **d**).

Neutrophil priming is usually associated with an increase in surface expression of opsonic receptors^38^. To investigate if GPR43 ligands also have such an influence, the abundance of complement and Fc receptors on neutrophils before and after acetate stimulation was compared. In contrast to the above-described assays, acetate led to upregulation of complement receptors CD11b (CR3) and CD35 (CR1), and of Fc receptor CD16 (FcyRIII) even in the absence of other MAMPs or endogenous GPCR agonists (Fig. 2a). This response could be blocked by CATPB indicating that it depended on GRP43 (Supplementary Fig. 2f).

Upregulation of opsonin receptors should increase the phagocytosis capacity of neutrophils. Indeed, 15-min pre-incubation of human neutrophils with acetate led to significantly enhanced phagocytosis of serum-opsonized *S. aureus* USA200 (Fig. 2b). This finding, together with the strongly increased oxidative burst suggested that GPR43 stimulation should improve the capacity of neutrophils to kill bacteria. Indeed, 10-min preincubation of neutrophils with acetate led to 25.7% stronger killing of serum-opsonized *S. aureus* (Fig. 2c)*.* Similar observations were made with the bacterial pathogens *Enterococcus faecalis*, *Listeria monocytogenesis*, and *Staphylococcus epidermidis* (Supplementary Fig. 2g) indicating that acetate priming has a general promoting impact on neutrophil phagocytosis. In agreement with the major role of NADPH oxidase in the antimicrobial activity of neutrophils, *S. aureus* killing was strongly reduced by treatment with the NADPH oxidase inhibitor diphenyleniodonium (DPI) (Supplementary Fig. 2h). When acetate was added to neutrophils at the same time as the opsonized bacteria or even 60 min later, improved killing could still be observed (Fig. 2d) suggesting that neutrophil priming via GPR43 can help to control invading pathogens even after the onset of an infection. *S. aureus* and several other bacterial species release high levels of acetate as an intermediary product of their energy metabolism^39^. When live *S. aureus* cells were co-cultivated with neutrophils for 3.5 h, they survived significantly better in the presence of the GPR43 inhibitor CATPB indicating that the sensing of *S. aureus*-produced acetate may be a prerequisite for efficient elimination of the bacteria by neutrophils (Fig. 2c). In agreement with the GPR43-dependence of acetate-promoted neutrophil *S. aureus* killing, acetate did not inhibit but even slightly improved growth of *S. aureus* (Supplementary Fig. 2i).

### Intraperitoneal injection of acetate increases serum acetate levels and primes neutrophils

**Fig. 3:**
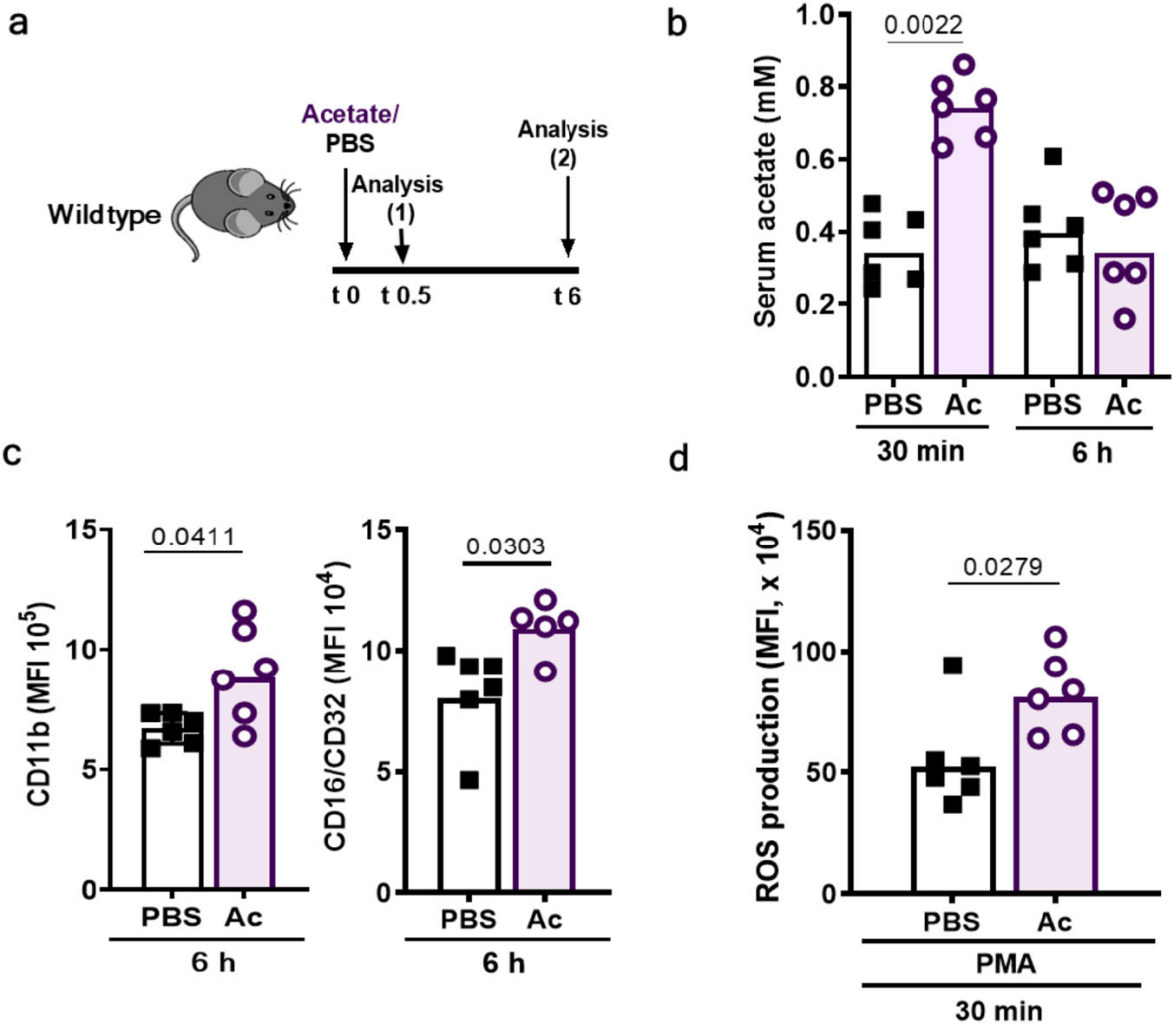
Intraperitoneal acetate injection increases serum acetate levels and primes neutrophils in mice. **a** Mice received an intra-peritoneal acetate (Ac) injection (500 mg/kg) followed by analysis of blood acetate concentrations (isolated from the tail vein) and neutrophil phenotypes 30 min and 6 h after injection. **b** Acetate (Ac) i.p. injection caused a two-fold serum acetate increase 30 min after injection and normal serum acetate levels were restored after 6 h. **c** Neutrophils isolated from mice treated with acetate showed enhanced complement (CD11b) and Fcγ (CD16/CD32) receptor expression compared to PBS-treated mice. **d** In vivo acetate (Ac) injection caused increased ROS production upon stimulation with phorbol-12-myristat-13-acetate (PMA). Data in all panels represent geometric means from six animals. *P < 0.05; **P < 0.01 significant difference versus the indicated condition as calculated by Mann–Whitney *U* test.

To analyze if acetate also primes neutrophils of mice in an in vivo setting, we injected mice intraperitoneally (i.p.) with acetate dissolved in PBS or with the same volume of PBS and analyzed blood acetate levels, neutrophil surface markers, and neutrophil capacity to generate ROS 6 h later (Fig. 3a). The serum of PBS-treated mice contained on average 341 ± 39.9 µM acetate. 30 min after i.p. injection of 500 mg/kg sodium acetate, the serum acetate concentration raised to 766 ± 34.8 µM (Fig. 3b). Neutrophils from acetate-treated mice showed enhanced expression of complement receptor CD11b (CR3) and Fc receptor CD16/32 (Fig. 3c) and significantly enhanced oxidative burst after stimulation with the synthetic protein kinase C activator phorbol-12-myristat-13-acetat (PMA) 6 h after infection compared to PBS-treated mice (Fig. 3d). Acetate had a significant impact on neutrophil opsonin receptor expression at 6 h after acetate injection although the serum acetate concentration had already normalized at this time point (Fig. 3b). Thus, mouse neutrophils are primed by acetate in a similar way as human neutrophils and maintain the primed state for several hours.

**Fig. 4:**
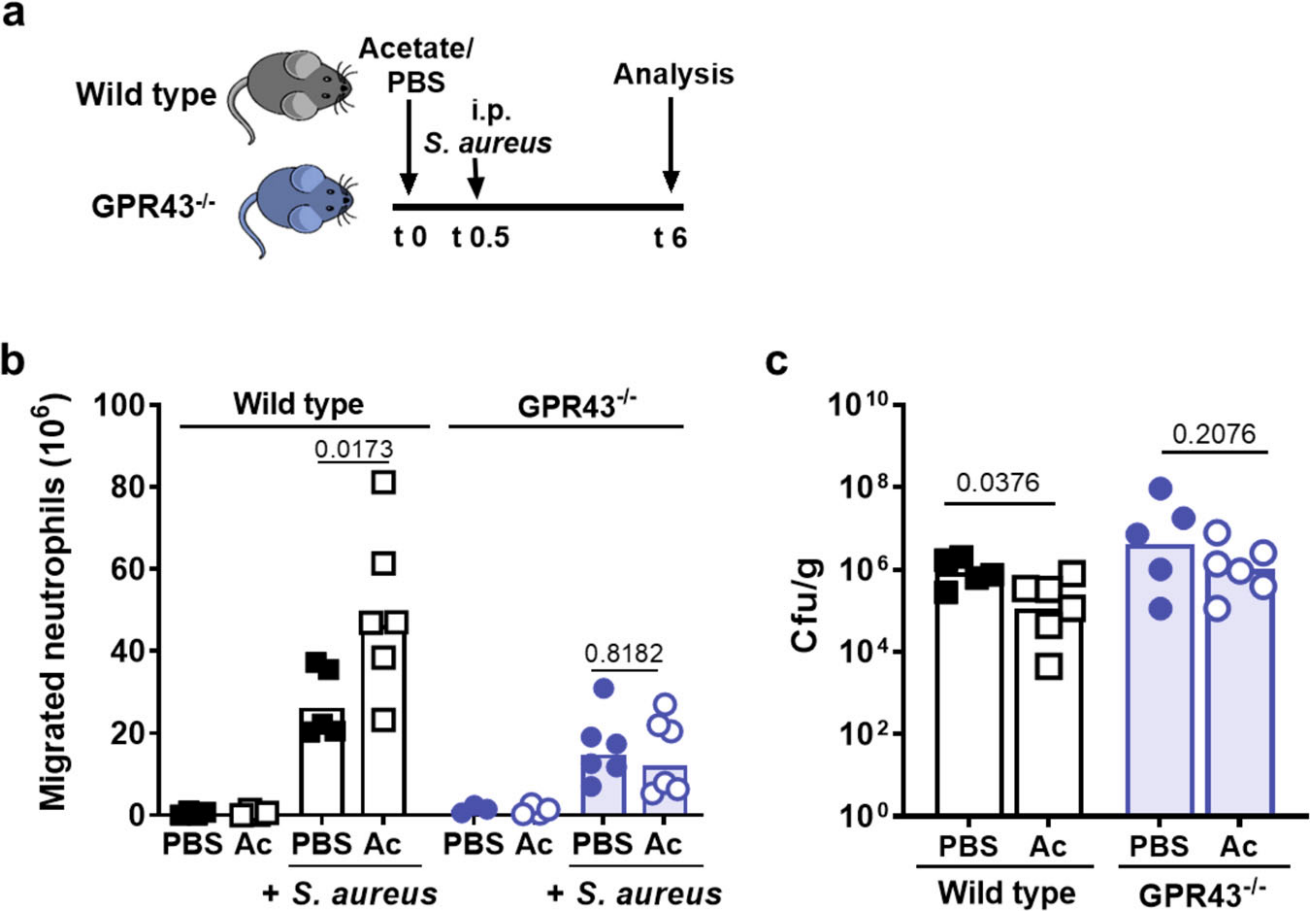
Intraperitoneal acetate injection enhances neutrophil influx and decreases bacterial loads in a mouse peritoneal infection model. **a** In the murine peritonitis model, mice were pre-treated with acetate (Ac) or PBS 30 min prior to intra-peritoneal *S. aureus* injection. **b** Acetate (Ac) treatment resulted in enhanced CD45^+^Ly6G^+^ neutrophil migration into the peritoneum compared to PBS-treated wild-type mice (black symbols) 6 h after infection. GPR43^−/−^ mice (blue symbols) showed reduced overall neutrophil migration into the peritoneum with no beneficial effect of acetate treatment. **c** Acetate-treated wild-type mice showed slightly reduced bacterial loads in the liver, whereas acetate treatment of GPR43^−/−^ mice did not influence bacterial loads. Data in panels **b** and **c** represent geometric means from six animals. *P < 0.05 significant difference versus the indicated PBS or wild-type control as calculated by Mann–Whitney *U* test.

Intraperitoneal acetate treatment did not alter the basal numbers of neutrophil and monocytes, neither in the blood nor in the peritoneum (Supplementary Fig. 3a–c). However, when mice were i.p.-infected by *S. aureus* USA200 30 min after the animals had received acetate rather than PBS i.p., neutrophil numbers in the peritoneum were significantly increased 6 h later (Fig. 4a, b). This difference was not observed in GPR43^−/−^ mice indicating that it resulted from GPR43-mediated acetate priming. Only a small fraction of the inoculated bacteria was recovered from the peritoneum after 6 h (Supplementary Fig. 3d), whereas the majority had spread to the liver (Fig. 4c) and other peripheral organs (Supplementary Fig. 3d). Wild-type and GPR43^−/−^ mice had comparable basic acetate concentrations in their sera (Supplementary Fig. 4a). PBS-treated wild-type mice had slightly higher peritoneal neutrophil numbers and lower *S. aureus* CFUs in the liver than GPR43^−/−^ mice (Fig. 4b, c), suggesting that the basal serum concentrations of acetate led to a weak priming level in the presence of a functional GPR43, which was strongly augmented by increasing the serum acetate concentration.

### GPR43-dependent acetate priming prevents severe courses of *S. aureus* sepsis

**Fig. 5:**
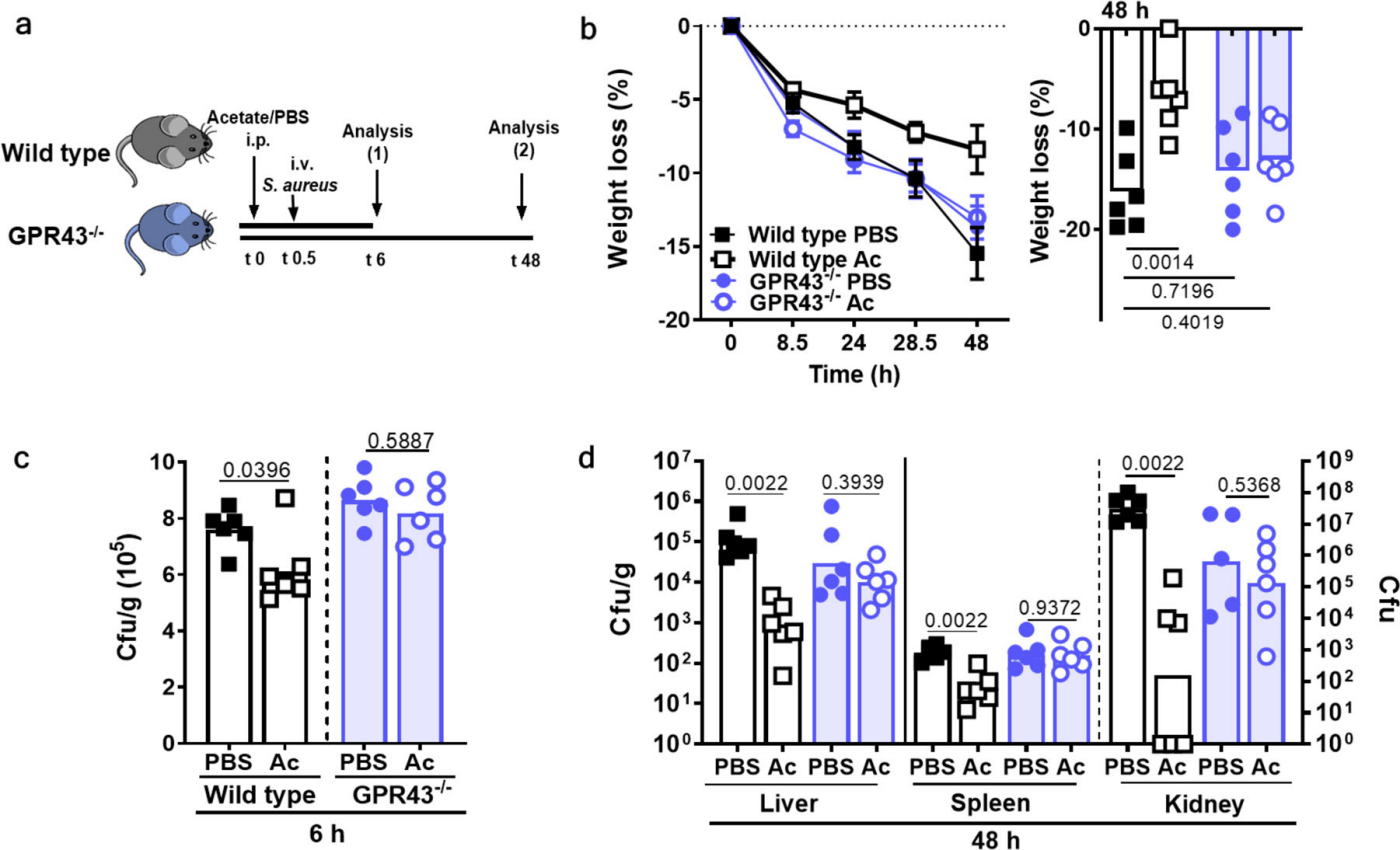
GPR43-dependent acetate priming is beneficial during *S. aureus* bloodstream infection. **a** Treatment of mice with acetate (Ac) 30 min prior to *S. aureus* i.v. injection resulted in **b** decreased weight loss in wild-type mice (black symbols) but not in GPR43^−/−^ mice (blue symbols). **c** Six hours after infection onset, bacterial loads were slightly reduced in the liver of acetate-treated wild-type mice (black), whereas acetate showed no such effect in GPR43^−/−^ mice (blue). **d** After 48 h, acetate (Ac) treatment prior to the onset of bloodstream infection resulted in drastically decreased bacterial loads in the liver, spleen, and kidney in wild-type mice, while no such difference was observed in GPR43^−/−^ mice (blue). Data in **b**–**e** panels represent geometric means or geometric means ± SEM (**b**) from six animals. *P < 0.05; **P < 0.01 significant difference versus the indicated PBS control as calculated by one-way ANOVA with Dunnett’s multiple comparisons test (**b**) and Mann–Whitney *U* test (**c** and **d**).

The finding that acetate levels and neutrophil priming state were increased after i.p. acetate injection on a systemic level raised the question, whether intraperitoneal acetate injection could also help to cure disseminated bacterial infections. *S. aureus* USA200 was injected into the bloodstream of wild-type and GPR43^−/−^ mice 30 min after intraperitoneal application of acetate or PBS (Fig. 5a). Whereas PBS-treated wild-type mice developed signs of severe disease and rapidly lost weight, the acetate-treated wild-type mice were less sick and lost much less weight. In contrast, treatment of GPR43^−/−^ mice with acetate did not influence weight loss (Fig. 5b, Supplementary Fig. 4b, c).

Six hours after infection, most of the injected bacteria were found in the liver, which is in agreement with previous studies demonstrating that the liver has a primordial role in early stages of *S. aureus* bloodstream infections. Notably, the bacterial numbers were significantly lower in the livers of acetate-treated wild-type mice, while acetate had no notable effect on the *S. aureus* population in GPR43^−/−^ mice indicating that GPR43 activation contributes to bacterial clearance from the bloodstream (Fig. 5c). At 48 h after infection, most of the bacteria had disappeared from the liver but the acetate-treated animals still had significantly lower *S. aureus* numbers compared to the mock-treatment in the liver (Fig. 5d). Whereas a similar situation was found in the spleen, the vast majority of the bacteria could be isolated from the kidneys. Here, the i.p. application of acetate led to a five-orders-of-magnitude reduced *S. aureus* density in wild-type animals (Fig. 5d). Again, acetate had no significant impact on bacterial numbers in GPR43^−/−^ mice indicating that the strong acetate-mediated improvement of the infection outcome depended on GPR43.

**Fig. 6:**
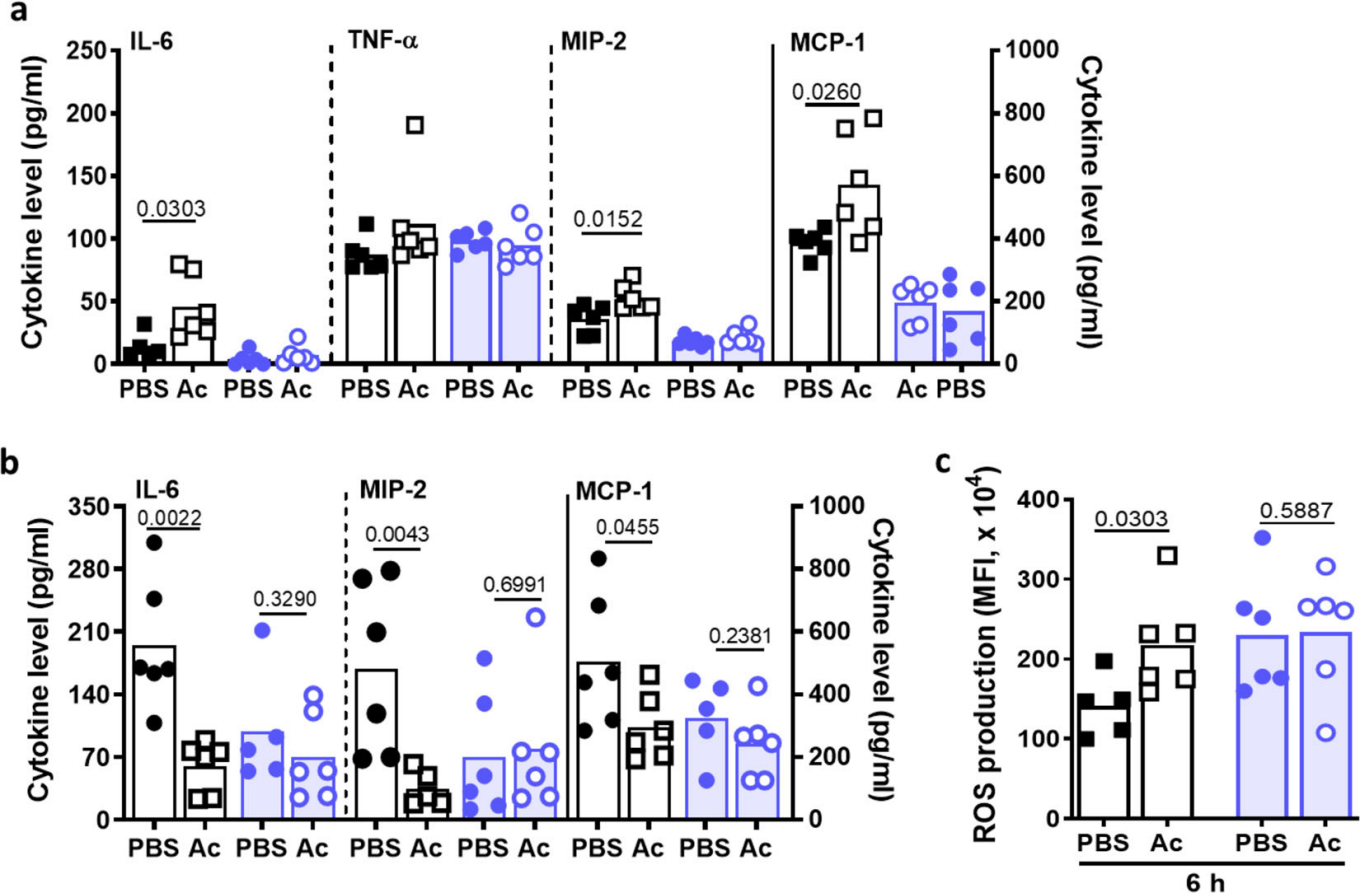
Intraperitoneal acetate priming promotes cytokine and ROS release during mouse bloodstream infection. **a** Treatment of wild-type mice (black symbols) with acetate prior to an *S. aureus* bloodstream infection, i.v. injection induces an initial increase in serum IL-6, MIP-2, and MCP-1 levels measured after 6 h. **b** This was accompanied by increased intrinsic ROS production by neutrophils from acetate-treated (Ac) wild-type mice, while acetate treatment had no such effect in GPR43^−/−^ mice (blue symbols). **c** 48 h after induction of a bloodstream infection, the cytokine levels in acetate-treated (Ac) wild-type but not in GPR43^−/−^ mice were significantly decreased compared to PBS-treated mice, which is in line with the decreased disease severity. Data in all panels represent geometric means from six animals. *P < 0.05; **P < 0.01 significant difference versus the indicated PBS control as calculated by Mann–Whitney *U* test.

I.p. acetate treatment had no effect on the basal cytokine levels 30 min or 6 h after application (Supplementary Fig. 5a, b). However, acetate-treated mice had slightly increased serum levels of the pro- and anti-inflammatory cytokine IL-6 (46 ± 10 µg/ml) and of the chemokines MIP-2 (CXCL2; 53 ± 4 µg/ml) and MCP-1 (CCL2; 590 ± 80 µg/ml) compared to PBS-treated mice 6 h after infection, probably as a consequence of the GPR43-dependent boost of anti-infective host defense (Fig. 6a). Likewise, neutrophils from acetate-treated mice exhibited increased intrinsic ROS production (Fig. 6c). In contrast, acetate application had no impact on serum cytokine and chemokine levels of GPR43^−/−^ mice (Fig. 6a). At 48 h after infection, the differences in cytokine patterns between acetate and PBS treatment had reversed. The recovery of acetate-treated wild-type animals was reflected by significantly lower concentrations of the cytokines IL-6 and TNF-α, two major mediators of sepsis-associated exuberant inflammation^7^, which were at high levels in the mock-treated wild-type animals (IL-6, 194,6 ± 29 µ/ml; TNF-α, 76 ± 10 µg/ml) (Fig. 6b, Supplementary Fig. 5c). Likewise, the chemokines MIP-2, MCP-1, and KC were reduced in acetate-treated wild-type animals. The lack of any significant differences in cytokine or chemokine levels between acetate or mock-treated GPR43^−/−^ mice confirmed that the presence of both, GPR43 and sufficient amounts of its agonist acetate, can prevent severe courses of sepsis in mice (Fig. 6b).

### Acetate improves sepsis outcome independently of the application route and even when applied after the onset of infection

To analyze if also oral application of acetate could improve sepsis outcome, acetate was added to the drinking water (neutralized to avoid rejection by mice), five days before mice were infected i.v. with *S. aureus* (Supplementary Fig. 6a). 48 h later we found significantly fewer bacterial numbers in the spleen of acetate-treated wild-type mice compared to the control mice. A similar trend was seen in the kidneys, which, however, did not reach significance (Supplementary Fig. 6b). Notably, oral acetate administration led to significantly reduced weight loss in the case of the acetate-fed wild-type mice, but no such influence of acetate on GPR43^−/−^ following infection by *S. aureus* compared to control-fed animals (Supplementary Fig. 6c, d). Only acetate-fed animals had increased serum acetate levels, which reached concentrations of 580 ± 150 µM (Supplementary Fig. 6e). These data indicate that acetate can prevent systemic *S. aureus* infections in similar ways if administered orally or intraperitoneally.

**Fig. 7:**
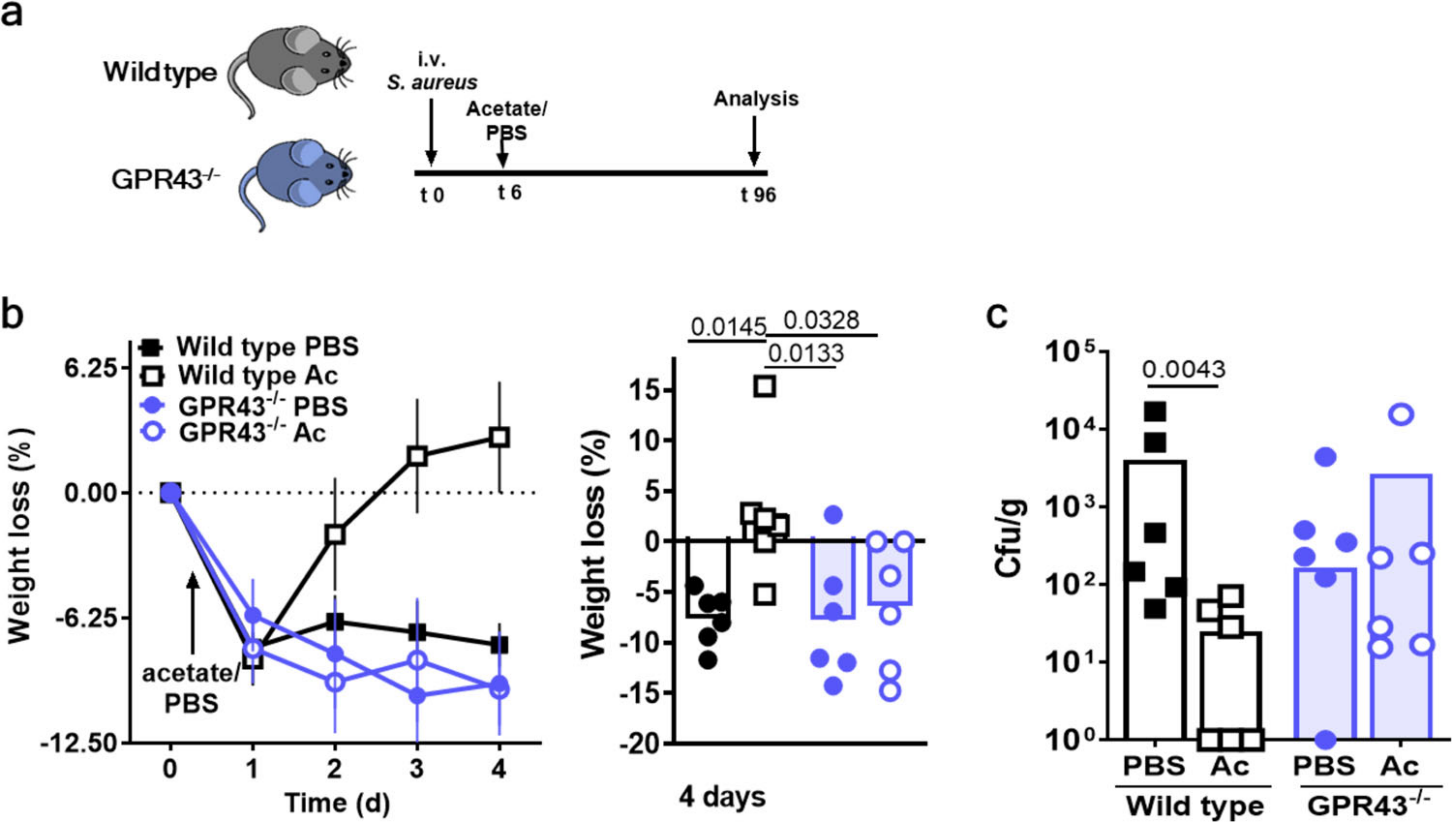
Acetate treatment after infection onset improves sepsis outcome. **a** Mice were treated with acetate (Ac) in PBS or an equal volume of PBS 6 h after the onset of an *S. aureus* bloodstream infection, and septic wild-type (black symbols) and GPR43^−/−^ (blue symbols) mice were monitored for 4 days. **b** Acetate-treated wild-type mice regained weight more rapidly than PBS-treated wild-type mice. **c** This was accompanied by a reduced bacterial load in the liver when compared to PBS-treated septic mice. GPR43^−/−^ mice displayed similar bacterial loads in the liver compared to PBS-treated wild-type mice (**c**). Acetate (Ac) treatment showed no effect in GPR43^−/−^ mice. Data in panels **b**, **c** represent geometric means or geometric means ± SEM (**b**) from six animals. *P < 0.05; **P < 0.01 significant difference versus the indicated condition as calculated by one-way ANOVA with Dunnett’s multiple comparisons test (**b**) or by Mann–Whitney *U* test (**c**).

The fact that the severe course of sepsis could be prevented by the presence of sufficiently high acetate concentrations in serum at the time point of *S. aureus* entry into the bloodstream raised the question, whether an interventional application of acetate after infection onset could still help to treat the disease. To assess this possibility wild-type and GPR43^−/−^ mice were i.v.-infected with *S. aureus* and were 6 h later treated via the intraperitoneal route with acetate or PBS (Fig. 7a). Already after two days of infection, the acetate treatment showed a slightly positive impact on the infection course (Supplementary Fig. 6f). To monitor the full course of infection, the infection period was extended to 4 days and a lower infection dose was used than in the previous experiments to avoid excessive weight loss and mortality. Indeed, acetate-treated wild-type mice lost weight only during the first day but rapidly gained weight at later time points after infection, whereas PBS-treated mice did not recover from sepsis (Fig. 7b). This was accompanied by decreased bacterial loads in the liver of acetate vs. PBS-treated mice at 4 days after infection (Fig. 7c). Together, these data indicate that the application of acetate could be an effective treatment option for sepsis even after the onset of an infection.

## Discussion

The pathophysiology of sepsis involves both, hyper-inflammatory and anti-inflammatory dysfunctions, which shape the course of the diseases in different phases and in different tissues. The dichotomy of these processes has made it extraordinarily difficult to identify suitable molecular targets for the prevention and therapy of sepsis and dozens of clinical trials with multiple immunomodulatory drugs have shown no efficacy or led even to aggravation of the disease^8^. Neutrophils are major players in the immunopathology of sepsis and either exuberant, persistent activation, or dampened, insufficient antimicrobial responses have been identified as major reasons for the failure of neutrophils to clear bloodstream infections^40,41^. Neutrophils can be stimulated by microbial or endogenous agonists in multiple ways, leading to different levels of priming or activation^42^. A high percentage of primed neutrophils can prevent bacterial infections even in neutropenic patients^43,44^. The extent and duration of priming may be crucial for the capacity of neutrophils to ameliorate or aggravate the disease. Our study demonstrates that activation of neutrophils via GPR43 leads to transient priming and improved capacities to ingest and kill bacterial invaders, which was reflected by substantially better resolution of sepsis in a mouse infection model. GPR43 has been extensively studied in the context of the intestinal microbiome–host interaction^45^ but has not been added to the list of potential targets for the treatment of sepsis. Nevertheless, a retrospective study has demonstrated that increased GPR43 expression in whole-blood samples of septic patients correlates with increased sepsis survival^23^, and GPR43 activation has been found to be beneficial for the treatment of lung infections^46,47^.

GPR43 seems to have a crucial role in neutrophil immune responses since it is highly expressed on human and mouse neutrophils, which distinguishes it from the two additional SCFA receptors GPR41 and GPR40^20^ that are predominantly found on enteroendocrine cells, enteric neurons, pancreatic beta cells, and in various regions of the human brain^48^. We demonstrate that acetate treatment primes neutrophils in a GPR43-dependent manner leading to enhanced neutrophil chemotaxis, bacterial killing as well as improved resolution of inflammation and sepsis outcome. Among the three GPR43 agonists, acetate, propionate, and butyrate, the only acetate can usually be found in the blood at concentrations near the effective concentration of ~0.5 mM^49^. However, serum acetate concentrations vary strongly and may lead to full GPR43 priming only in certain instances. Nevertheless, even average serum acetate amounts may cause a basal level of neutrophil priming even in the absence of acetate injection. In accord with this assumption, we found that wild-type mice had a general tendency to cope better with *S. aureus* sepsis than GPR43^−/−^ mice. Increasing the serum acetate concentration by i.p. injection or addition to the drinking water of sodium acetate had a transient, yet profound impact on neutrophil ROS production, serum cytokine levels, and bacterial clearance in peripheral organs. Some incidental reports support the positive role of acetate in the control of infections. Local SCFA treatment has been found to reduce the diameter of MRSA skin abscesses in mice^50^. Moreover, clinical data have shown that septic patients treated with volume substitutes that contain acetate show less mortality than patients treated with substitutes lacking acetate^51^.

Multiple processes can influence the concentration of acetate in human serum and other body fluids. The major acetate sources seem to be food intake and intestinal bacterial microbiome members, which produce SCFAs as fermentation products^52^. Under certain conditions such as phases of high alcohol consumption^26^, starvation, or diabetes also the liver produces acetate^48^. Moreover, some pathogens including *S. aureus* produces acetate during substrate-level phosphorylation in case of carbon overflow^53,54^, suggesting that GPR43 might activate neutrophils in response to local bacterial metabolic activity. A recent study found that neutrophils from GPR43^−/−^ mice were less capable of clearing bacterial pneumonia than wild-type mice even when the animals had not been treated with acetate^46^. It is possible that the local acetate release by the pathogen^55^ and specific dietary conditions led to a sufficiently high acetate concentration in the lung to allow GPR43-dependent host defence even in the absence of interventional acetate treatment while during systemic infection the bacteria-produced acetate cannot accumulate locally.

Our study indicates that the severity of sepsis in mice depends critically on transient neutrophil priming, which can be shaped by elevating serum acetate concentrations. Acetate had a strong positive impact on *S. aureus* sepsis if applied before or after the onset of infection and even if it was orally applied, indicating that it could be used in a prophylactic or therapeutic fashion. A recently reported synthetic GPR43 agonist that enhanced the response of human neutrophils to acetate could become an attractive lead compound for future GPR43-targeting drugs^56^. Since acetate priming enabled neutrophils to better eliminate several unrelated bacteria, GPR43-based interventions might be of help for a wide range of sepsis-causing pathogens. It has been shown that the intestinal microbiome compositions play a central role in sepsis^57^. One of the crucial hallmarks of a health-promoting microbiome dominated by Firmicutes and Bacteroidetes is the production of high amounts of SCFA such as acetate^58,59^. The combined effects of artificial nutrition and sustained exposure to antibiotics in severely ill patients can lead to the disruption of a health-promoting gut microbiome^60^. Fecal microbiome transplantation (FMT) as a potential therapy could be an option. However, such an approach needs extreme caution as a recent report demonstrated the transmission of a multi-drug-resistant organism via FMT, which subsequently caused lethal bacteremia^61^. The precise optimization of microbiome composition to increase the content of SCFA-producing commensals would be preferable but will require extensive research efforts.

Our data suggest that the direct application of acetate to patients receiving enteral nutrition or i.v. in case of parenteral nutrition could become a therapeutic option also in non-invasive ways by supplying acetate-rich food or a fiber-rich diet, which enhances the production of SCFAs by fermenting gut microbiota members such as Firmicutes and Actinobacteria species^62^.

## Methods

### Material

Sodium acetate (Sigma Aldrich) stock solution was pH-adjusted to 7.2 ± 0.5 in order to avoid unspecific cellular responses to altered pH values. Formylated PSMα3 (fMEFVAKLFKFFKDLLGFLGNN) peptide was kindly provided by S. Stevanović (University Tubingen). N-Formyl-Met-Leu-Phe (fMLF) and phorbol 12-myristate 13-acetate (PMA) were purchased from Sigma Aldrich, P_2_CysK_4,_ and LPS from Invivogen, rhC5a from R&D, and platelet-activating factor (PAF) from Biomol. The GPR43 agonist 4-CMTB and the GPR43 antagonist CATPB were synthesized by EMC (Tübingen).

### Bacteria

*S. aureus* strain USA200 (UAMS-1) was kindly provided by K. Bayles (University of Nebraska)^34^. *L. monocytogenes* (ATCC19118), *E. faecalis* (BK4684)^63^, and *S. epidermidis* (Tü3298)^64^ were utilized for neutrophil killing experiments. All bacteria were inoculated at OD_600_ 0.1 in tryptic soy broth (TSB) or lysogeny broth (LB, only *S. epidermidis*) and grown for 4 h under aerobic conditions (medium to flask ratio 1:5) followed by three washing steps with PBS. For optimal recognition by neutrophils, bacteria were opsonized with 10% pooled normal human serum (NHS) for 60 min and, if not otherwise mentioned, bacteria and neutrophils were used at a ratio of 1:1 (MOI of 1). Bacterial culture filtrates were obtained by centrifugation and subsequent filtration through 0.2-µm pore size filters (Merck).

### Neutrophil isolation

Human neutrophils were isolated by standard ficoll/histopaque gradient centrifugation^14^. If not otherwise mentioned neutrophils were suspended in RPMI with 5% human serum albumin, 2% HEPES, and 1% pyruvate. For inhibition of GPR43, neutrophils were incubated with 2.5 μM CATPB for 10 min. Blood was kindly donated by healthy volunteers (age 20–50) upon informed consent.

### ROS production and chemotaxis

ROS production by human neutrophils was measured over a time period of one hour by monitoring luminol-amplified chemiluminescence using 282 µM luminol (Sigma Aldrich). If not otherwise mentioned, neutrophils were incubated with 1 mM acetate for 10 min and subsequently stimulated with fMLF (500 nM), PSMα3 (500 nM), PAF (2 µM), C5a (100 ng/ml), or opsonized live *S. aureus* bacteria (MOI 2).

For the analysis of the chemotactic capacities of different stimuli, neutrophils were loaded with 3 µM 2′,7′-bis-(2-carboxyethyl)-5-(and-6)-carboxyfluorescein, acetoxymethyl ester (BCECF-AM, Molecular Probes). The migration along gradients of the indicated stimuli was monitored using 3-µm polycarbone trans-well membranes (Greiner). The chemotactic index was calculated as the percentage of total cells migrated to the lower chamber and corrected by the buffer control. PSMα3 and fMLF were applied at concentrations of 375 and 10 nM, respectively, and live opsonised *S. aureus* USA200 with an MOI of 1. The GPR43 agonist 4-CMTB (EMC, Tübingen) was used at a final concentration of 1 μM.

### Western blot

Neutrophils were incubated with the indicated acetate concentrations for 15 min followed by lysis with immunoprecipitation (IP)-lysis buffer (ThermoFisher). The subsequent immunoprecipitation was performed using the Dynabead Protein G IP Kit (ThermoFisher). Briefly, a mouse anti-human p47^phox^–specific monoclonal antibody (BD Bioscience) was bound to the protein G beads for 15 min under constant shaking (350 rpm). After washing, dynabeads were added to the cell lysis and incubated for 15 min followed by washing, addition of elution and SDS–PAGE sample buffer, and denaturation for 5 min at 99 °C. Samples were subjected to a standard 4–20%-SDS–PAGE gel (BioRad). Proteins were blotted to a nitrocellulose membrane and p47^phox^ and p47^phox^ S345 were visualized using mouse anti-human p47^phox^ (BD) and rabbit anti-human p47phox S345 (Invitrogen) antibodies. Secondary antibodies were IRDye 680CW anti-rabbit and IRDye 800CW anti-mouse from Licor. Protein bands were detected with the Licor Odyssey CLx.

### Surface receptor analysis and phagocytosis

Neutrophils were stimulated for 60 min with 1 mM acetate, followed by 45 min staining with antibodies directed against the different surface receptors. As a positive control for analysis of IL-8 receptor downregulation, 250 nM fMLF was used. Anti-CD35-PE (clone E11, Miltenyi), anti-CD16-PE (clone 3G8, BD Bioscience), anti-CD11b-PE (clone ICRF44, BD Bioscience), anti-hCXCR1/IL8-RA-PE (clone 42705, R&D), and anti-hCXCR2-RB-PE (clone 242216, R&D) were used at a 1:40 dilution (Supplementary Fig. 7a). Prior to measurement with the FACSCalibur (BD), neutrophils were fixed with 3.7% formaldehyde for 20 min.

For the phagocytosis assay, bacteria from an overnight culture were washed and stained with 10 µM CFSE (Sigma Aldrich) for 60 min. CFSE-stained bacteria were opsonized for 60 min with 10% human pooled serum (University Hospital Tübingen). Neutrophils were pre-incubated with the indicated acetate concentration for 30 min before incubation with bacteria at an MOI of 1 for a further 30 min. Neutrophils were fixed with 3.7% formaldehyde for 20 min prior to measurement with the FACSCalibur (BD). This assay measures in principle both, extracellular, neutrophil-adherent and intracellular bacteria. However, our previous control experiments using an agent that quenches extracellular fluorescence have demonstrated that the vast majority of neutrophil-associated opsonized *S. aureus* are indeed intracellular after only a few seconds^65^.

### Bacterial killing

Bacteria from a 4-h culture grown in TSB were washed and opsonized with 10% pooled human serum (University Hospital Tübingen) for 60 min at 37 °C. Neutrophils were stimulated with the indicated concentration of acetate or buffer for 30 min prior to or post-incubation with bacteria (MOI of 1). For inhibition of the NADPH oxidase, neutrophils were pre-incubated with 5 µM dibenziodolium chloride (DPI) for 20 min prior to incubation with bacteria. The numbers of surviving bacteria were detected by determination of the colony-forming units (CFUs) per ml. Bacterial survival was calculated in relation to a bacterial control without neutrophils.

### Cytokine detection

The release of IL-8 from neutrophils was measured with a human IL-8/CXCL8 ELISA Kit (R&D). Primary neutrophils were stimulated with 1 or 10 mM acetate 30 min prior to incubation with the indicated secondary stimuli for a further 4.5 h. Stimuli were used at the following concentrations: PSMα3 500 nM; fMLF 500 nM; P_2_Cysk_4_ 200 ng/ml; LPS 100 nM; opsonized USA200 bacteria at MOI of 1. Human IL-8 detection in the cellular supernatant was performed according to the IL-8 ELISA vendor’s manual. Cytokines in mouse serum were detected using the Bioplex Mouse Cytokine Assay (BioRad) and BioRad Multiplex Instrument.

### Mouse infection assay

All experimental procedures involving mice were carried out according to protocols approved by the Animal Ethics Committees of the Regierungspräsidium Tübingen (IMIT1/17 and IMIT1/18). Gpr43^−/−^ mice were kindly provided by Stephan Offermanns and have been previously described^66^ and bred in the animal facility of the University Hospital of Tübingen. C57BL/6N mice (Envigo/Netherlands) were used as wild-type control mice. Mice were bred under specific pathogen-free conditions under 22 °C, 50–55% relative humidity, and 12 h/12 h light/dark cycle conditions.

For in vivo analysis of the acetate effect on neutrophils, 6–8 weeks-old female C57BL/6N mice were i.p. injected with 500 mg/kg sodium acetate in PBS (pH 7.2) or with PBS (pH 7.2). 30 min after injection, blood was drawn to analyze serum acetate concentrations, blood leukocyte counts, cytokine levels, and ROS production by leukocytes. ROS production was determined in whole blood by staining with 5 μM DCFDA (Sigma Aldrich) for 10 min prior to erythrocyte lysis using a solution of 155 mM NH_4_Cl, 10 mM KHCO_3_, and 0.1 mM EDTA with a pH of 7.4. After DCFDA incubation, neutrophils were stimulated with RPMI or 200 nM PMA for 10 min. Serum acetate levels were measured using the acetate colorimetric assay kit (Sigma Aldrich). Blood was centrifuged for 10 min with 500×*g* to obtain mouse serum. In order to decrease cell debris, the mouse sera were cleared using a 10-kDa centrifugation cartridge. The acetate measurement was performed according to the vendors’ instructions. Blood leukocyte counts were determined by antibody staining and subsequent flow cytometry using a FACS LSR Fortessa X-20 (BD). Neutrophils were stained with Ly6G (APC, clone REA526) and CD45 (APC or PE, clone REA737, monocytes with CD14 (PE, clone REA934) and CD45 (APC, clone REA526) (all Miltenyi). For the cytokine detection, mouse blood was obtained by cardiac puncture and incubation with heparin for 30 min. Serum was gained by centrifugation and was rapidly frozen at −80 °C before analysis with a Bio-plex Mouse Cytokine Assay (BioRad) according to the vendor’s instruction. Briefly, mouse sera were diluted 1–4 and measured using a Bio-plex Pro Mouse Cytokine 8-plex Assay kit including TNF-alpha, MIP-2, MCP-1, and IL-6.

Six hours after acetate or PBS treatment, mice were euthanized and the surface receptor expression of peripheral blood leukocytes was determined. For this purpose, erythrocytes were lysed and leukocytes were stained with monoclonal antibodies specific for mouse CD45, Ly6G (APC, clone REA526), CD14 (PE, clone REA934), CD16/32 (PE, clone REA370), or CD11b (PE, clone REA592) (all Miltenyi). Ly6G was used as a neutrophil marker (Supplementary Fig. 7b). The staining was analyzed by a FACS LSR Fortessa X-20 (BD).

In the mouse sepsis model, 1 × 10^7^ (48 h) or 2.5 × 10^6^ (4 days) colony-forming units (cfu) of *S. aureus* USA200 were injected intravenously into 6–8 weeks old female C57BL/6N wild-type or GPR43^−/−^ mice. Acetate treatment occurred 30 min prior or 6 h after *S. aureus* infection by i.p. injection of 500 mg/kg sodium acetate in PBS or the same volume of PBS. Six hours, 48 h, or 4 days after infection, mice were euthanized with CO_2_. Subsequently, CFUs in organs were determined by plating serial dilutions on agar plates and leukocytes were stained for ROS production and receptor expression as described above. Leukocyte staining was determined with a FACS LSR Fortessa X-20 (BD). For the cytokine detection mouse serum was obtained and rapidly stored at −80 °C before analysis with a Bioplex Mouse Cytokine Assay (BioRad) according to vendors’ instruction.

For the mouse peritonitis model, 6–8-weeks-old female C57BL/6N wild-type and GPR43^−/−^ mice were treated with 500 mg/kg acetate in PBS or PBS. 30 min after treatment, 5 × 10^8^ CFUs of *S. aureus* USA200 were injected in the peritoneum. At 6 h after infection mice were euthanized with CO_2_ and peritoneal exudates were collected and leukocytes stained as described above. The numbers of immigrated cells were detected by counting in the peritoneal exudates.

For the non-invasive acetate application model, 6–8 weeks-old female C57BL/6N wild-type and GPR43^−/−^ mice were fed for 7 days with 150 mM sodium acetate (pH 7.3) or 150 mM sodium chloride (control) in drinking water. Mice were given ad libidum access to drinking water. Five days after treatment begins, 1 × 10^7^ CFUs of *S. aureus* USA200 were injected i.v. to induce sepsis. 48 h or 4 days after infection, mice were euthanized with CO_2_. Subsequently, *S. aureus* CFUs in organs were determined by plating serial dilutions on agar plates.

### Statistics and reproducibility

Statistical analysis was performed using Graph Pad Prism 8.0. (GraphPad Software, La Jolla, USA). Normal distributions were analyzed by two-tailed Student’s *t* test or Mann–Whitney *U* test to compare two data groups unless otherwise stated. For comparison of more than two data groups, one-way ANOVA with Dunnett’s multiple comparisons test was used. For each figure, the replicate number *n* is indicated in the figure legend and represents the number of independently performed biological replicates.

### Reporting summary

Further information on research design is available in the Nature Research Reporting Summary linked to this article.

## Supplementary information

Supplementary Information

Reporting Summary

Description of Supplementary Files

Supplementary Data 1

## Data Availability

The authors declare that the main data supporting the findings of this study are available within the article and its Supplementary Information files (Supplementary Data 1). All other data are available from the corresponding author on reasonable request.
