# Supplementary material for: Acetate sensing by GPR43 alarms neutrophils and protects from severe sepsis
Source: Commun Biol. 2021 Jul 30;4:928. doi: 10.1038/s42003-021-02427-0 (PMC8324776; doi:10.1038/s42003-021-02427-0)
Supplement: Supplementary file 1 — Supplementary Information [file 42003_2021_2427_MOESM1_ESM.pdf]

Acetate sensing by GPR43 alarms neutrophils and protects from severe sepsis

Katja Schlatterer[1,2,3], Christian Beck[1,2,3], Ulrich Schoppmeier[4,2,3], Andreas Peschel[1,2,3]*, Dorothee Kretschmer[1,2,3]

[1] Interfaculty Institute for Microbiology and Infection Medicine Tübingen (IMIT), Infection Biology, University of Tübingen, Auf der Morgenstelle 28, 72076 Tübingen, Germany

[2] German Center for Infection Research, partner site Tübingen, Germany

[3] Cluster of Excellence EXC 2124 Controlling Microbes to Fight Infections

[4] Interfaculty Institute for Microbiology and Infection Medicine Tübingen (IMIT), Medical Microbiology and Hygiene, University of Tübingen, Elfriede-Aulhorn-Straße 6, 72076 Tübingen, Germany

Supplementary Materials

Suppl. Figure 1: GPR43 activation increases oxidative burst in human neutrophils.

Suppl. Figure 2: In-vitro impact of GPR43 activation on chemotaxis, receptor expression, cytokine secretion, and bacterial elimination.

Suppl. Figure 3: Immunological characteristics of wild-type and GPR43-/- mice 30 minutes after intraperitoneal acetate treatment.

Suppl. Figure 4: Sepsis-induced weight loss in acetate-treated mice and bacterial counts in mouse peritoneum and spleen upon intraperitoneal acetate application.

Suppl. Figure 5: Blood cytokines in mouse serum upon intraperitoneal acetate application.

Suppl. Figure 6: Sepsis-induced weight loss and bacterial burden in mouse spleen and kidneys after oral administration of acetate.

Suppl. Figure 7: Gating strategies for flow cytometric analysis of human and mouse neutrophils.

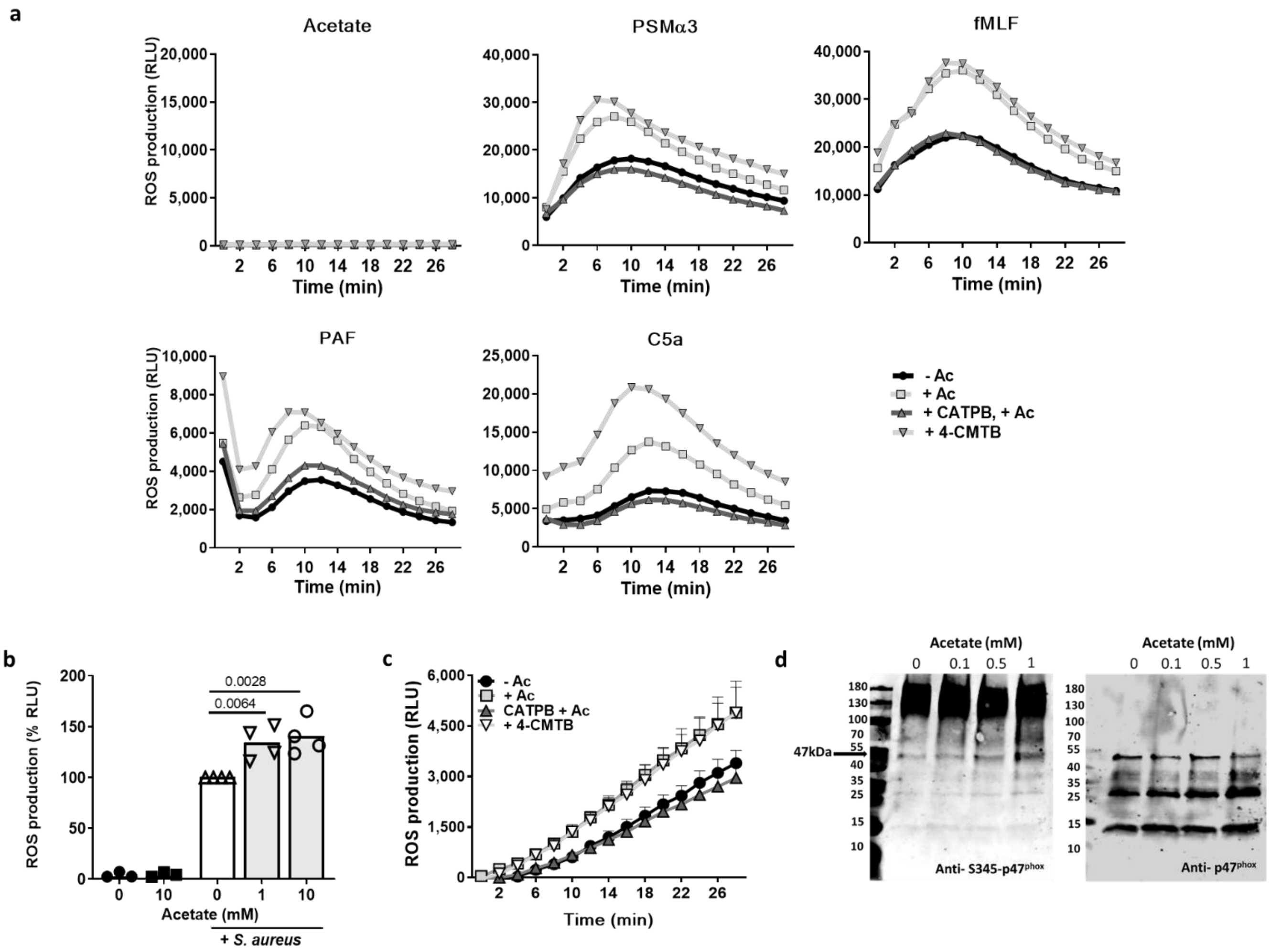

Suppl. Figure 1: GPR43 activation increases oxidative burst in human neutrophils.

(a) The oxidative burst induced by the bacteria-derived fMLF (500 nM), PSMα3 (500 nM), host-derived GPCR ligands PAF (2 μM), or C5a (100 ng/ml) or (b, c) by *S. aureus* cells (MOI 2) was enhanced by acetate pre-treatment, which could be inhibited by the GPR43 antagonist CATPB. The synthetic agonist 4-CMTB mimicked the effect of acetate, which was completely reversible by addition of CATPB. (d) Western blot-based detection of phosphorylated p47^{phox} (S345) and p47^{phox} in neutrophils treated with the indicated acetate concentration for three minutes. P47^{phox} and the phosphorylation site S345 were visualized on the same gel using mouse anti-human p47^{phox} and rabbit anti-human p47^{phox} S345 antibodies and the respective IRDye secondary antibodies. P47^{phox} has a size of 47 kDa and appears between the size marker for 55 and 40 kDa. Gel pictures are unprocessed as gained on the Licor Odyssey CLx. Data in panels a-c represent means or means ± SEMs (c) from n=4 (b)

and n=5 (a, c) independent experiments. d shows one representative western blot. P values represent significant difference versus the indicated condition as calculated by one-way ANOVA with Dunnett's multiple comparisons test (a).

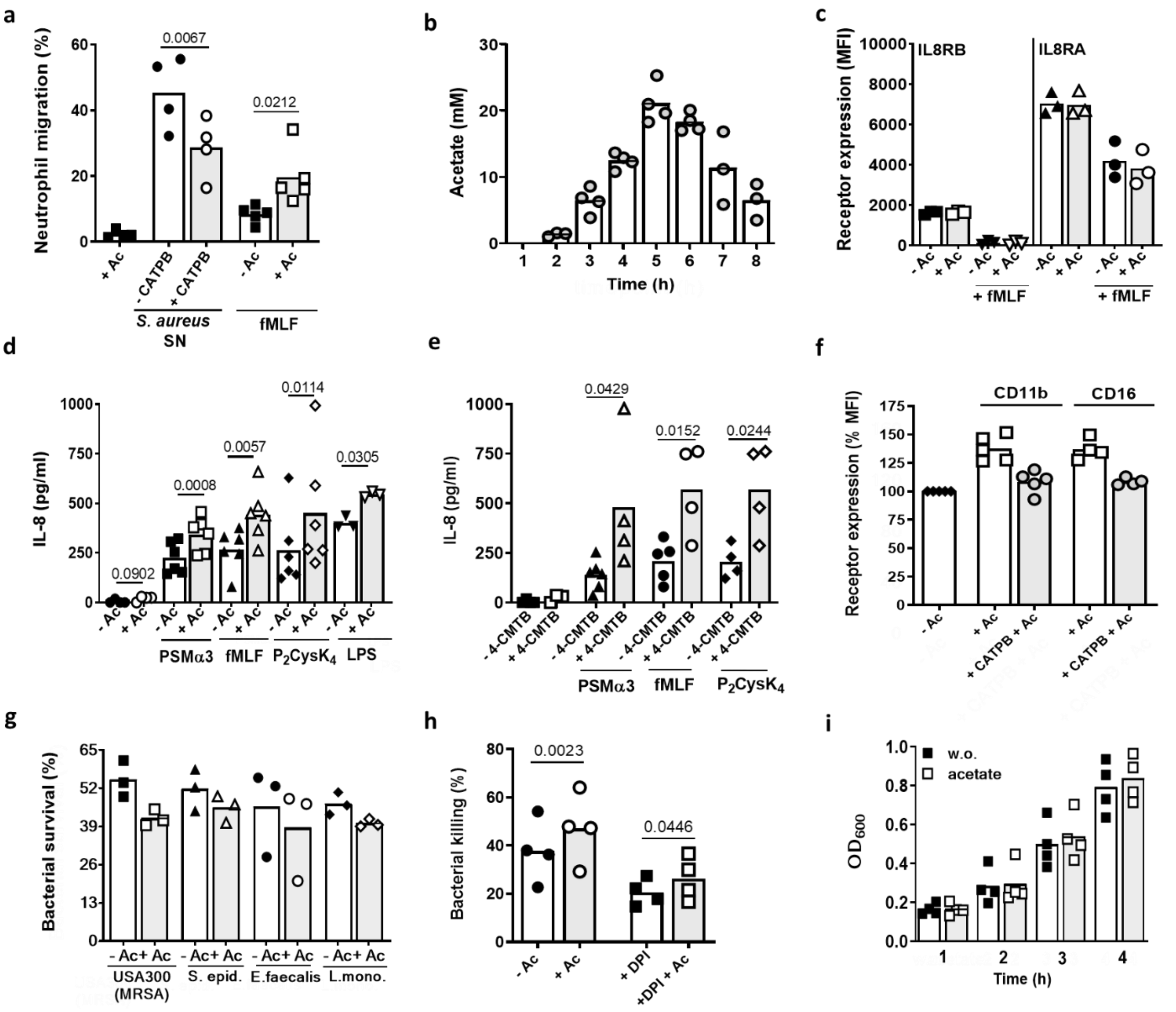

Suppl. Figure 2: *In-vitro* impact of GPR43 activation on chemotaxis, receptor expression, cytokine secretion, and bacterial elimination.

(a) GPR43 inhibition by CATPB decreased the migration of neutrophils towards *S. aureus* culture filtrates (10-fold diluted) and pre-incubation of neutrophils with 1 mM acetate enhanced the migration towards 10 nM of the FPR1 ligand fMLF (b) *S. aureus* produced up to 20 mM acetate during cultivation in broth**.** (c) Expression of the IL-8 receptors IL8RB and IL8RA is not affected by acetate treatment (1mM). (d/ e) Neutrophil priming with acetate or the synthetic GPR43 agonist 4-CMTB increased IL-8 secretion following stimulation by the FPR2 ligand PSMα3 (500 nM), the FPR1 ligand fMLF (500 nM), the TLR2 ligand P_2CysK_4 (200 ng/ml), or the TLR4 ligand LPS (100 nM). (f) The acetate-mediated enhanced expression of complement receptor CD11b and Fc receptor CD16 could be prevented by CATPB indicating that it is GPR43-

dependent. (g) Acetate-treated neutrophils showed increased killing of serum-opsonized *S. aureus* USA300 (MRSA), *S. epidermidis*, *E. faecalis,* and *L. monocytogenes*. (h) Inhibition of the NADPH oxidase by dibenziodolium chloride (DPI) decreased the positive effect of acetate on the bacterial killing but did not abolish it. (i) Supplementation of growth media with 10 mM acetate showed no inhibitory effect on bacterial growth. Data in panels a, b, e, h, i represent means from n=4, in panel c and d represents means from n=3 and from panel f and d represents means from n=5 independent experiments. The indicated p values represent significant difference versus the negative control as calculated by paired two-tailed Student’s t-test.

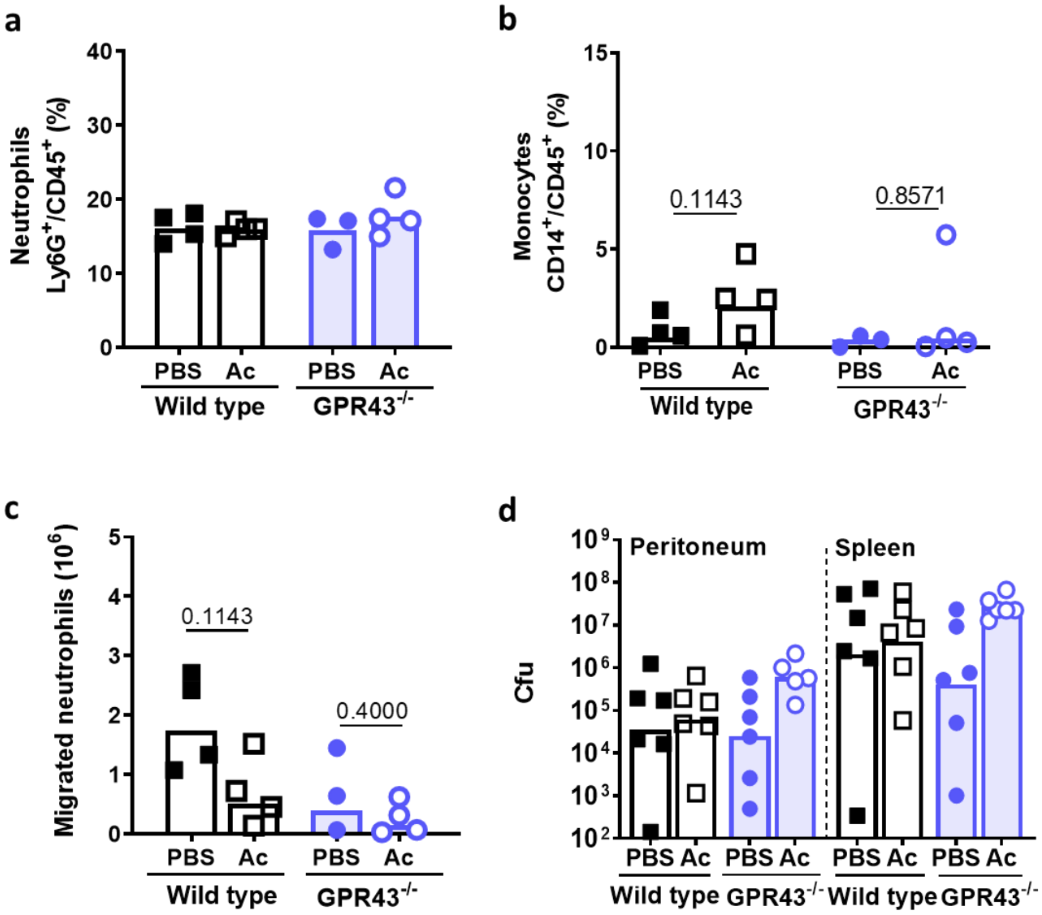

Suppl. Figure 3: Immunological characteristics of wild-type and GPR43$^{-/-}$ mice 30 minutes after intraperitoneal acetate treatment.

Percentage of blood (a) Ly6G^{+}/CD45^{+} neutrophils or (b) CD14^{+}/CD45^{+} monocytes in intraperitoneally PBS or acetate-treated wild-type and GPR43$^{-/-}$ mice. (c) Neutrophil numbers in the peritoneal cavity 30 minutes after acetate (Ac) or PBS injection. (d) Serum acetate levels in wild-type or GPR43$^{-/-}$ mice 30 minutes after PBS treatment (blood isolated by cardiac puncture). (d) *S. aureus* loads recovered from peritoneum and spleen at six hours after infection in a peritonitis model. Data in all panels represent geometric means from four (a –c) or six (d) animals per group. P values represent significant difference versus the PBS control as calculated by Mann-Whitney Test.

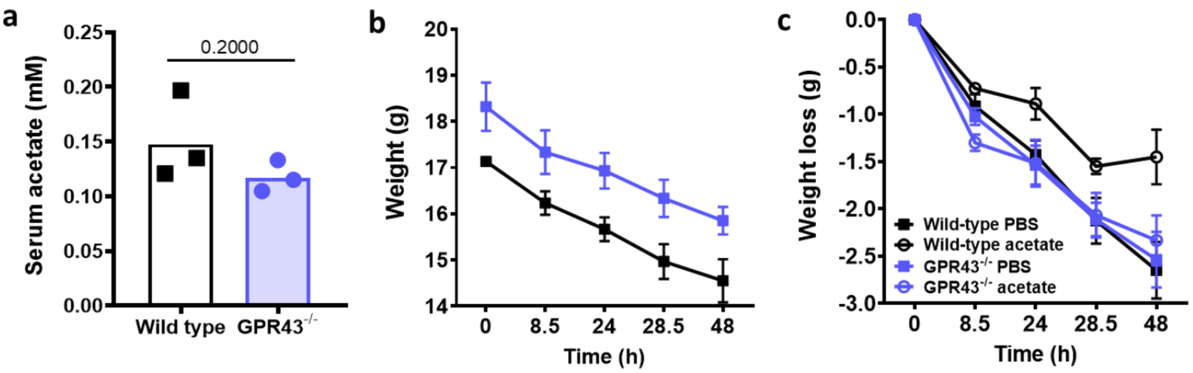

Suppl. Figure 4: Sepsis-induced weight loss in acetate-treated mice and bacterial counts in mouse peritoneum and spleen upon intraperitoneal acetate application.

(a) Total body weight (in gram) in wild-type and GPR43$^{-/-}$ mice after intravenous *S. aureus* injection. At similar age, GPR43$^{-/-}$ mice have a higher basal weight compared to wild-type mice. (b) Comparison of sepsis-induced weight loss (in gram) in acetate- or PBS-treated wild-type or GPR43$^{-/-}$ mice showed similar weight loss between PBS-treated wild-type and GPR43$^{-/-}$ mice. Data in all panels represent geometric means of three (a) or geometric means ± SEM or six mice (b, c) per group. P values represent significant difference versus the indicated PBS control as calculated by Mann-Whitney-U test.

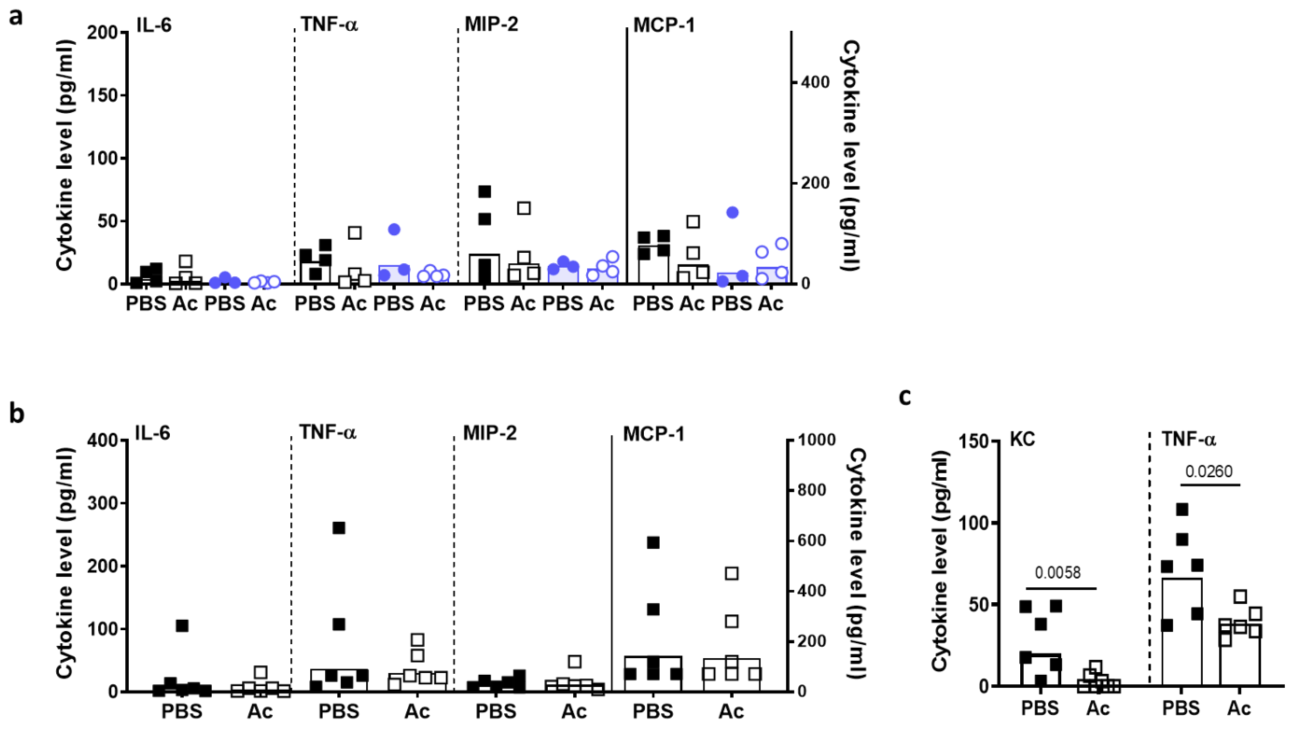

Suppl. Figure 5: Blood cytokines in mouse serum upon intraperitoneal acetate application.

(a) 30 minutes after acetate or PBS injection, blood cytokine levels in wild-type and GPR43 knockout mice showed no difference. (b) No difference in blood cytokine levels was observed in wild-type mice, six hours after acetate or PBS injection. (c) Acetate-treated wild-type mice showed reduced cytokine (KC, TNF-α) levels 48 hours after the onset of a *S. aureus* bloodstream infection. Data in all panels represent means of four (a) or six (b) mice per group. *, $P < 0.05$ difference versus the indicated PBS control as calculated by Mann-Whitney-U test.

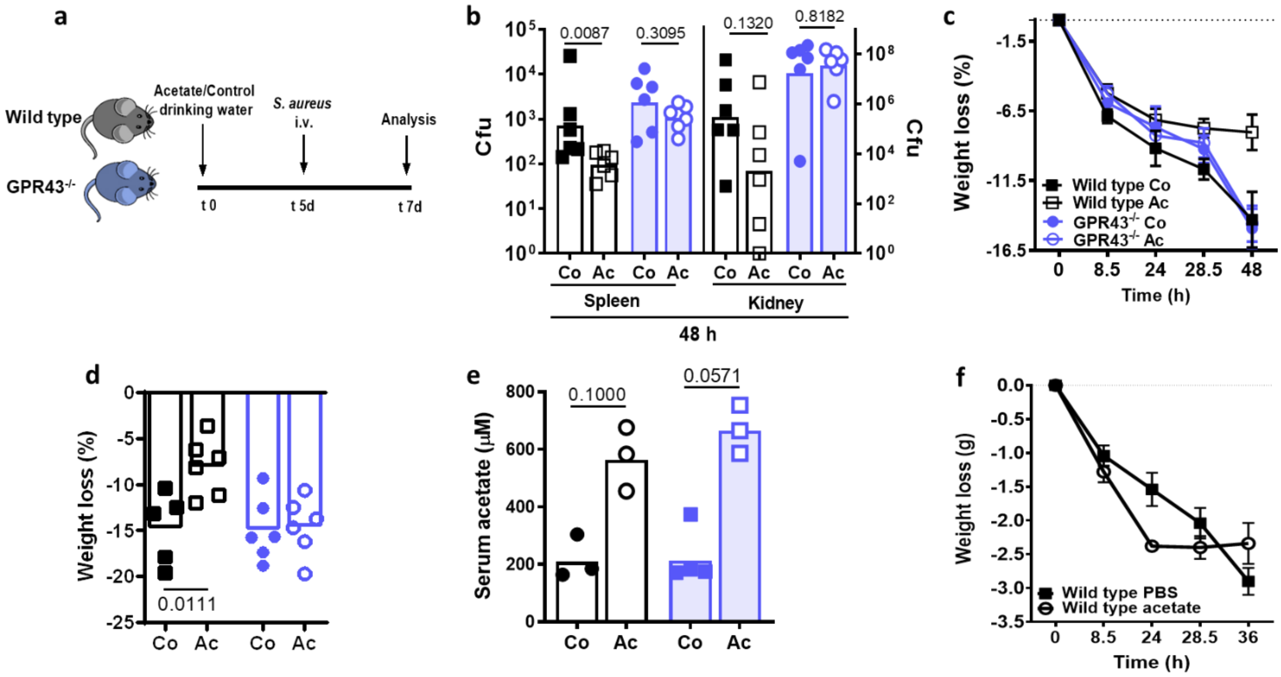

Suppl. Figure 6: Sepsis-induced weight loss and bacterial burden in mouse spleen and kidneys after oral administration of acetate.

(a) Wild-type or GPR43$^{-/-}$ mice were fed for seven days with 150 mM sodium acetate (Ac) or sodium chloride (Co) in the drinking water. Five days after treatment start, *S. aureus* sepsis was induced and bacterial loads were analysed 48 hours later. Acetate-treated wild-type mice showed significantly reduced bacterial loads in the spleen (b), which was accompanied by significantly reduced weight loss after 48 hours (c, d). (e) Application of sodium acetate in the drinking water resulted in an increased serum acetate level in wild-type and GPR43 knockout mice (blood isolated by cardiac puncture) measured 48 h after sepsis onset. (f) Intraperitoneal acetate injection 6 hours post-sepsis induction with 10^7 cfu influence weight loss in wild-type mice. Data in figure e represent geometric means of three or four mice per group. Data in all other panels represent geometric means or geometric means ± SEM (c, f) of six mice per group. P values represent significant difference versus the indicated control (Co) as calculated by Mann-Whitney-U test.

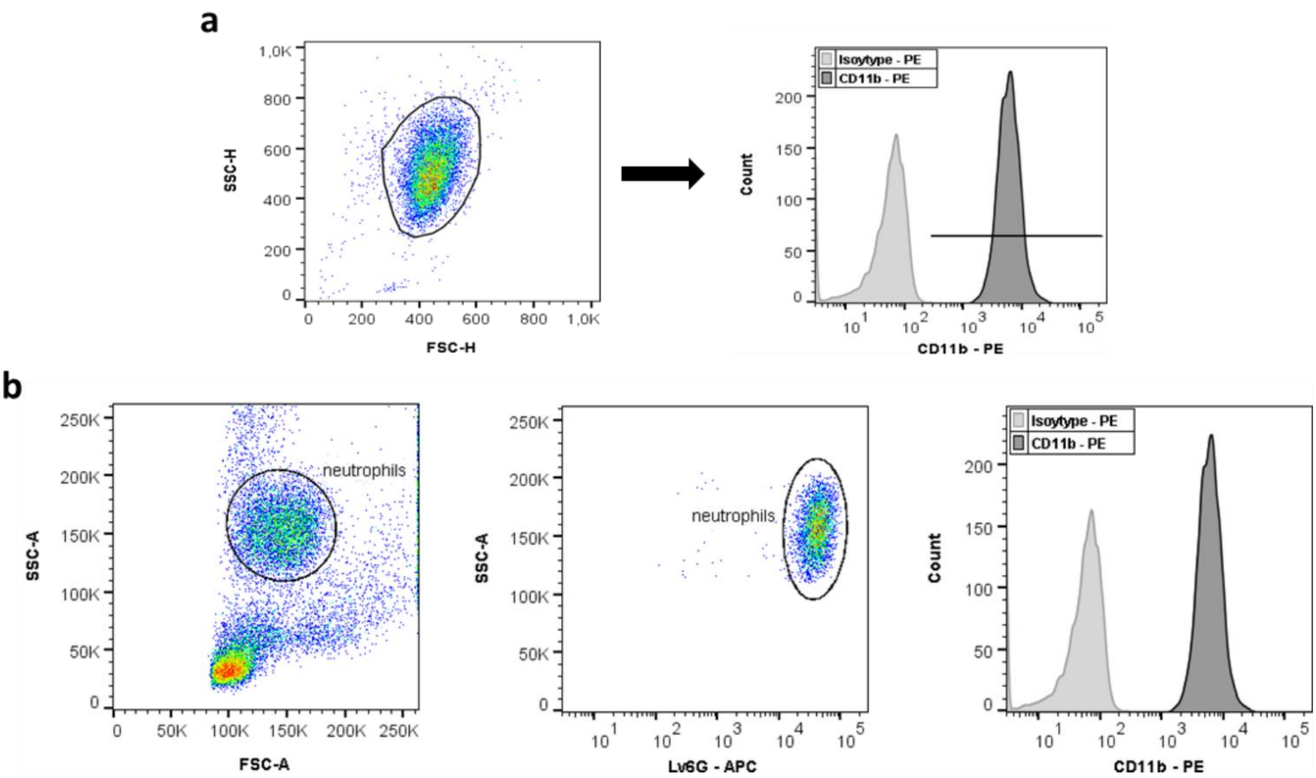

Suppl.Figure 7: Gating strategies for flow cytometric analysis of human and mouse neutrophils.

(a) Gating strategy for human neutrophil assays. Neutrophils were separated by Histopaque/Ficoll gradient and subsequent gating of neutrophils occurred at the FSC/SCC density plot according to size and granularity (left). Histopaque/Ficoll gradient isolations showed a neutrophil purity of more than 80%. With the help of isotype or negative controls and the FL2 (PE) or FL1 (CFSE) fluorescence channel, the gated population was subdivided into fluorescence-positive and -negative cells (right). The receptor expression or phagocytic capacity was expressed as product of the mean fluorescence of the fluorescence-positive population and their relative abundance (mean fluorescence intensity, MFI). (b) Gating strategy for mouse neutrophil assays. Whole mouse blood was erythrocyte-lysed and subsequent neutrophil gating occurred at the FSC/SCC density according to size and granularity (left) and with Ly6G – allophycocyanin (APC) staining (middle). With the help of isotype or negative controls and the FL2 (PE) or FL1 (DCFDA) fluorescence channel, the gated population was subdivided into fluorescence-positive and -negative cells (right). The receptor expression or ROS production was expressed as product of the mean fluorescence of the fluorescence-positive population and their relative abundance (mean fluorescence intensity, MFI).
