# Supplementary material for: Acetate sensing by GPR43 alarms neutrophils and protects from severe sepsis
Source: Commun Biol. 2021 Jul 30;4:928. doi: 10.1038/s42003-021-02427-0 (PMC8324776; doi:10.1038/s42003-021-02427-0)
Supplement: Supplementary file 2 — Reporting Summary [file 42003_2021_2427_MOESM2_ESM.pdf]

nature research

Corresponding author(s): Andreas Peschel, Katja Schlatterer, Dorothee Kretschmer, Christian Beck, Ulrich Schoppmeier

Last updated by author(s): Jun 21, 2021



# Reporting Summary

Nature Research wishes to improve the reproducibility of the work that we publish. This form provides structure for consistency and transparency in reporting. For further information on Nature Research policies, see our Editorial Policies and the Editorial Policy Checklist.

## Statistics

For all statistical analyses, confirm that the following items are present in the figure legend, table legend, main text, or Methods section.

| n/a | Confirmed | |
|---|---|---|
| ☐ | ☒ | The exact sample size (*n*) for each experimental group/condition, given as a discrete number and unit of measurement |
| ☐ | ☒ | A statement on whether measurements were taken from distinct samples or whether the same sample was measured repeatedly |
| ☐ | ☒ | The statistical test(s) used AND whether they are one- or two-sided<br>*Only common tests should be described solely by name; describe more complex techniques in the Methods section.* |
| ☐ | ☒ | A description of all covariates tested |
| ☐ | ☒ | A description of any assumptions or corrections, such as tests of normality and adjustment for multiple comparisons |
| ☐ | ☒ | A full description of the statistical parameters including central tendency (e.g. means) or other basic estimates (e.g. regression coefficient) AND variation (e.g. standard deviation) or associated estimates of uncertainty (e.g. confidence intervals) |
| ☒ | ☐ | For null hypothesis testing, the test statistic (e.g. *F*, *t*, *r*) with confidence intervals, effect sizes, degrees of freedom and *P* value noted<br>*Give P values as exact values whenever suitable.* |
| ☒ | ☐ | For Bayesian analysis, information on the choice of priors and Markov chain Monte Carlo settings |
| ☒ | ☐ | For hierarchical and complex designs, identification of the appropriate level for tests and full reporting of outcomes |
| ☒ | ☐ | Estimates of effect sizes (e.g. Cohen's *d*, Pearson's *r*), indicating how they were calculated |

*Our web collection on statistics for biologists contains articles on many of the points above.*

## Software and code

Policy information about availability of computer code

| | |
|---|---|
| Data collection | No software was used to collect the data. |
| Data analysis | Flow Jo, Statistical analysis was performed by using GraphPad Prism (GraphPad419 Software, Inc)Version 8 |

For manuscripts utilizing custom algorithms or software that are central to the research but not yet described in published literature, software must be made available to editors and reviewers. We strongly encourage code deposition in a community repository (e.g. GitHub). See the Nature Research guidelines for submitting code & software for further information.

## Data

Policy information about availability of data

All manuscripts must include a data availability statement. This statement should provide the following information, where applicable:

- Accession codes, unique identifiers, or web links for publicly available datasets
- A list of figures that have associated raw data
- A description of any restrictions on data availability

All major data generated or analysed in this study are included in the article or its supplementary information files. All other data relating to this study are available from the corresponding author on reasonable request.

# Field-specific reporting

Please select the one below that is the best fit for your research. If you are not sure, read the appropriate sections before making your selection.

☒ Life sciences ☐ Behavioural & social sciences ☐ Ecological, evolutionary & environmental sciences

For a reference copy of the document with all sections, see nature.com/documents/nr-reporting-summary-flat.pdf

# Life sciences study design

All studies must disclose on these points even when the disclosure is negative.

| | |
|---|---|
| Sample size | Sample sizes of in vitro and in vivo experiments were chosen to obtain significant results. The prospective power analysis was performed with appropriate statistical methods. |
| Data exclusions | No data points from in vitro or in vivo experiments were excluded. |
| Replication | Each major experiment was repeated at least three times as stated in the manuscript. For the immunoblot analysis a typical experiment representative of 3 independent experiments is shown as stated in the figure legend. |
| Randomization | Human donors were randomly selected for blood donation and mice were grouped randomly. |
| Blinding | Blinding was not necessary in our study. |

# Reporting for specific materials, systems and methods

We require information from authors about some types of materials, experimental systems and methods used in many studies. Here, indicate whether each material, system or method listed is relevant to your study. If you are not sure if a list item applies to your research, read the appropriate section before selecting a response.

## Materials & experimental systems

| n/a | Involved in the study | |
|---|---|---|
| ☐ | ☒ | Antibodies |
| ☒ | ☐ | Eukaryotic cell lines |
| ☒ | ☐ | Palaeontology and archaeology |
| ☐ | ☒ | Animals and other organisms |
| ☐ | ☒ | Human research participants |
| ☒ | ☐ | Clinical data |
| ☒ | ☐ | Dual use research of concern |

## Methods

| n/a | Involved in the study | |
|---|---|---|
| ☒ | ☐ | ChIP-seq |
| ☐ | ☒ | Flow cytometry |
| ☒ | ☐ | MRI-based neuroimaging |

## Antibodies

| | |
|---|---|
| Antibodies used | human CD11b-PE (clone ICRF44), human CD16-PE (clone 3G8) (both BD Bioscience, 557321 and 555407), human CD35-PE (clone E11, Miltenyi 130-124-236), anti-mouse CD11b-PE (clone REA592, 130-113-806), anti-mouse CD16/CD32-PE (clone REAL370, 130-120-533), anti-mouse Ly6G-APC (clone REA526, 130-120-734), anti-mouse CD14-PE (clone REA934, 130-115-558), anti-mouse CD21/CD35 (clone REA800, 130-111-730) (all Miltenyi),anti-hCXCR1/IL8-RA-PE (R&D),anti-hCXCR2-RB-PE (R&D) |
| Validation | antibodies are validated by the respective companies (BD Bioscience, Milteny, R& D) |

## Animals and other organisms

Policy information about studies involving animals; ARRIVE guidelines recommended for reporting animal research

| | |
|---|---|
| Laboratory animals | 6 to 8 weeks-old femal wild type C57BL/6N mice and GPR43-/- C57BL/6N mice were used. |
| Wild animals | The study did not involve wild animals. |
| Field-collected samples | The study did not involve field-collected animals or organisms. |
| Ethics oversight | All experimental procedures involving mice were carried out according to protocols approved by the Animal Ethics Committees of the Regierungspräsidium Tübingen (IMIT1/17 and IMIT1/18). |

Note that full information on the approval of the study protocol must also be provided in the manuscript.

## Human research participants

Policy information about studies involving human research participants

| | |
|---|---|
| Population characteristics | Individual neutrophil donors were healthy volunteers selected randomly irrespective of sex and age. |
| Recruitment | Donors of neutrophils were healthy volunteers selected randomly irrespective of sex. |
| Ethics oversight | Neutrophil isolation from peripheral blood of healthy human volunteers was approved by the Ethics Committees of the University of Tübingen |

Note that full information on the approval of the study protocol must also be provided in the manuscript.

## Flow Cytometry

### Plots

Confirm that:

☒ The axis labels state the marker and fluorochrome used (e.g. CD4-FITC).

☒ The axis scales are clearly visible. Include numbers along axes only for bottom left plot of group (a 'group' is an analysis of identical markers).

☐ All plots are contour plots with outliers or pseudocolor plots.

☐ A numerical value for number of cells or percentage (with statistics) is provided.

### Methodology

| | |
|---|---|
| Sample preparation | Isolated human neutrophils or mouse leukocytes were stained with the respective antibodies prior to cell fixation. |
| Instrument | BD FACS Calibur (model number 342976), BD FACS LSRFortessa X-20 |
| Software | CellQuest Pro Version 5, BD FACS Diva Version 8, FlowJo V10 |
| Cell population abundance | Neutrophils were isolated prior to analysis. Neutrophil isolates contained approximatly 80-90 % intact neutrophils,which were separated during measurement from cell debris/erythrocytes via FSC/SSC gating.<br>Gated mouse neutrophils had an abundance of 10-15 % in the leukocyte isolate and a purity above 80 %. |
| Gating strategy | Human neutrophils were isolated by Histopaque-Ficoll-gradient centrifugation and contaminating erythrocytes were lysed prior to analysis. Due to individual donor characteristics the samples contained an average of approximatly 80-90 % intact neutrophils,which were separated during measurement from cell debris/erythrocytes via FSC/SSC gating.<br>Mouse blood was subjected to erythrocyte lysis and the remaining leukocytes were stained with the neutrophil marker Ly6G-APC and the monocyte or macrophage marker CD14-PE or F4/80 Vio770 to enable neutrophil gating. Additionally, leukocytes were stained with the B-cell marker CD21/CD35 PE and CD11b to support neutrophil gating. Gated neutrophils had an abandance of 10-15% and a purity above 80%. |

☒ Tick this box to confirm that a figure exemplifying the gating strategy is provided in the Supplementary Information.
