# Supplementary material for: Acetate sensing by GPR43 alarms neutrophils and protects from severe sepsis
Source: Commun Biol. 2021 Jul 30;4:928. doi: 10.1038/s42003-021-02427-0 (PMC8324776; doi:10.1038/s42003-021-02427-0)
Supplement: Supplementary file 3 — Description of Supplementary Files [file 42003_2021_2427_MOESM3_ESM.pdf]

Description of additional Supplementary Files

File name: Supplementary Data 1
Description: Source data of main figures.
